# Myeloid lineage cells evince distinct steady-state level of certain gene groups in dependence on hereditary angioedema severity

**DOI:** 10.3389/fgene.2023.1123914

**Published:** 2023-07-04

**Authors:** Lucie Ballonová, Přemysl Souček, Peter Slanina, Kamila Réblová, Ondřej Zapletal, Marcela Vlková, Roman Hakl, Viktor Bíly, Hana Grombiříková, Eliška Svobodová, Petra Kulíšková, Julie Štíchová, Marta Sobotková, Radana Zachová, Jana Hanzlíková, Martina Vachová, Pavlína Králíčková, Irena Krčmová, Miloš Jeseňák, Tomáš Freiberger

**Affiliations:** ^1^ Centre for Cardiovascular Surgery and Transplantation, Brno, Czechia; ^2^ Department of Experimental Biology, Faculty of Science, Masaryk University, Brno, Czechia; ^3^ Faculty of Medicine, Masaryk University, Brno, Czechia; ^4^ Central European Institute of Technology, Masaryk University, Brno, Czechia; ^5^Department of Allergology and Clinical Immunology, St. Anne’s University Hospital in Brno, Brno, Czechia; ^6^Department of Immunology, Second Medical School Charles University and University Hospital Motol, Brno, Czechia; ^7^Department of Immunology and Allergology, University Hospital Pilsen, Pilsen, Czechia; ^8^ Department of Immunology and Allergology, Faculty of Medicine in Pilsen, Charles University, Pilsen, Czechia; ^9^ Institute of Clinical Immunology and Allergy, University Hospital Hradec Kralove, Charles University, Faculty of Medicine in Hradec Kralove, Hradec Kralove, Czechia; ^10^ National Centre for Hereditary Angioedema, Department of Pediatrics, Jessenius Faculty of Medicine, Comenius University in Bratislava, University Teaching Hospital in Martin, Martin, Slovakia; ^11^Department of Pulmonology and Phthisiology, Jessenius Faculty of Medicine, Comenius University in Bratislava, University Teaching Hospital in Martin, Martin, Slovakia; ^12^ Depatment of Clinical Immunology and Allergology, Comenius University in Bratislava, Comenius University in Bratislava, University Teaching Hospital in Martin, Martin, Slovakia

**Keywords:** FXII, hereditary angioedema, immune cell, interferon-gamma, gene expression

## Abstract

Hereditary angioedema (HAE) is a rare genetic disorder with variable expressivity even in carriers of the same underlying genetic defect, suggesting other genetic and epigenetic factors participate in modifying HAE severity. Recent knowledge indicates the role of immune cells in several aspects of HAE pathogenesis, which makes monocytes and macrophages candidates to mediate these effects. Here we combined a search for HAE phenotype modifying gene variants with the characterization of selected genes’ mRNA levels in monocyte and macrophages in a symptom-free period. While no such gene variant was found to be associated with a more severe or milder disease, patients revealed a higher number of dysregulated genes and their expression profile was significantly altered, which was typically manifested by changes in individual gene expression or by strengthened or weakened relations in mutually co-expressed gene groups, depending on HAE severity. *SERPING1* showed decreased expression in HAE-C1INH patients, but this effect was significant only in patients carrying mutations supposedly activating nonsense-mediated decay. Pro-inflammatory CXC chemokine superfamily members *CXCL8, 10* and *11* were downregulated, while other genes such as *FCGR1A*, or long non-coding RNA *NEAT1* were upregulated in patients. Co-expression within some gene groups (such as an NF-kappaB function related group) was strengthened in patients with a severe and/or mild course compared to controls. All these findings show that transcript levels in myeloid cells achieve different activation or depression levels in HAE-C1INH patients than in healthy controls and/or based on disease severity and could participate in determining the HAE phenotype.

## 1 Introduction

Hereditary angioedema (HAE) is a life-threatening inborn error of immunity characterized by recurrent attacks of swelling caused by local bradykinin overproduction, increased vascular permeability and subsequent angioedema development. HAE is clinically a very heterogeneous disease affecting various organs with various intensities (Bork et al., 2006).

The high disease manifestation heterogeneity is observed despite the primary genetic reason being mostly obvious, residing in inactivating mutations in *SERPING1* (HAE-C1INH, types I and II), or *FXII* (HAE with normal C1 inhibitor, nC1-INH HAE). In very rare cases, *PLG, KNG1, ANGPT1, MYOF* and *HS3ST6* are suspected of being involved in nC1-INH HAE type pathogenesis (Bafunno et al., 2018; Bork et al., 2018; Dewald, 2018; Bork et al., 2019; Ariano et al., 2020; Bork et al., 2021), although further evidence is needed to approve their causal role, in particular, in the latter two. However, genetic determinants of disease severity remain unclear. It is dependent neither on the *SERPING1* mutation type (Germenis and Speletas, 2016), nor on the C1 inhibitor function which indicates the involvement of other factors (Drouet et al., 2008). The plasma contact (kallikrein-kinin) activation system releases bradykinin, a vasoactive inflammatory mediator triggering angioedema attacks, which makes it a candidate for such a disease-modifying effect. For example, the presence of a C allele (c.-4 T>C variant) in the FXII polymorphic nucleotide was associated with earlier disease onset (Speletas et al., 2015). Similarly, kallikrein variant KLKB1-428G was significantly associated with a delay in disease manifestation (Gianni et al., 2017). Although not associated with a particular gene variant, Drouet et al. (2008) observed lower APP (aminopeptidase P, an enzyme degrading bradykinin) activity in patients with severe HAE. Similarly, angiopoetin-2 and VEGF-A (Loffredo et al., 2016), or MASP-1 plasma levels (Bo Hansen et al., 2015) were higher in patients with a more severe HAE phenotype. Despite these observations, it is hardly possible to explain the overall HAE variable expressivity with these changes only.

The endothelial barrier, a site where local bradykinin production leads to increased vascular permeability, has a crucial role in edema development (Wu et al., 2020) and modulators of its function are suspected to be involved in HAE pathophysiology. Vascular permeability is also regulated by different immune cells including monocytes and macrophages (Rodrigues and Granger, 2015; Ferrara et al., 2021). Macrophages can serve as a cell surface for contact system component assembly producing an appreciable amount of active kinins and thus serve as a supplementary kinin source besides the endothelial surface (Barbasz and Kozik, 2009). While it is well known that the bradykinin system is involved in blood pressure control (Sharma, 2009), and some cells like monocyte-derived dendritic cells can interact with it through bradykinin receptor expression (Bertram et al., 2007), monocyte/macrophage participation in angioedema attacks has not been clearly demonstrated.

Nevertheless, some results show that these immune cells could play an important role in HAE development. Kallikrein can stimulate pro-inflammatory gene expression such as TNF, or IL-6 in monocytes (Yang et al., 2017) and applying bradykinin receptor BKR1 agonist stimulates monocyte migration (Hillmeister et al., 2011). Secreted phospholipases A2 (sPLA2) level is increased in patients’ plasma in a symptom-free period (Loffredo et al., 2018), which can subsequently induce VEGF-A and VEGF-C production in macrophages (Granata et al., 2010), and other vascular permeability modulating growth factors angiopoietins (Ang1, 2) follow the same trend. This trend seems to be more pronounced for VEGF-A and Ang2, factors inducing higher vascular permeability, in patients with more severe HAE (Loffredo et al., 2016). Additionally, the expression profile of monocytes/macrophages can be modulated by treating with bradykinin (Guevara-Lora et al., 2009), or cleaved kininogen (a molecule released after bradykinin cleavage) (Khan et al., 2006).

To uncover factors modifying HAE severity, we combined a DNA-based search of gene variants in candidate genes potentially involved in HAE pathogenesis with the characterization of monocyte/macrophage populations on the transcriptomic level in a symptom-free period.

## 2 Results

### 2.1 HAE patients’ classification

Patients suffering from HAE-C1INH all display different disease severity in terms of age at onset, attack frequency and localization, but also show different progress during aging and treatment. For the purposes of our investigation, we had to exactly define particular patient subgroups according to their disease course. In general, we prioritized some characteristics over other previously used approaches (Freiberger et al., 2011; Forjaz et al., 2021; Parsopoulou et al., 2022), for example, we decided not to consider an attack localization. While the attack localization affects HAE severity from the clinical point of view, especially laryngeal attacks threatening the patients’ life, we considered the genetic determination of age at onset and number of episodes of edema to be more clearly determined compared with attack localization.

**FIGURE 1:**
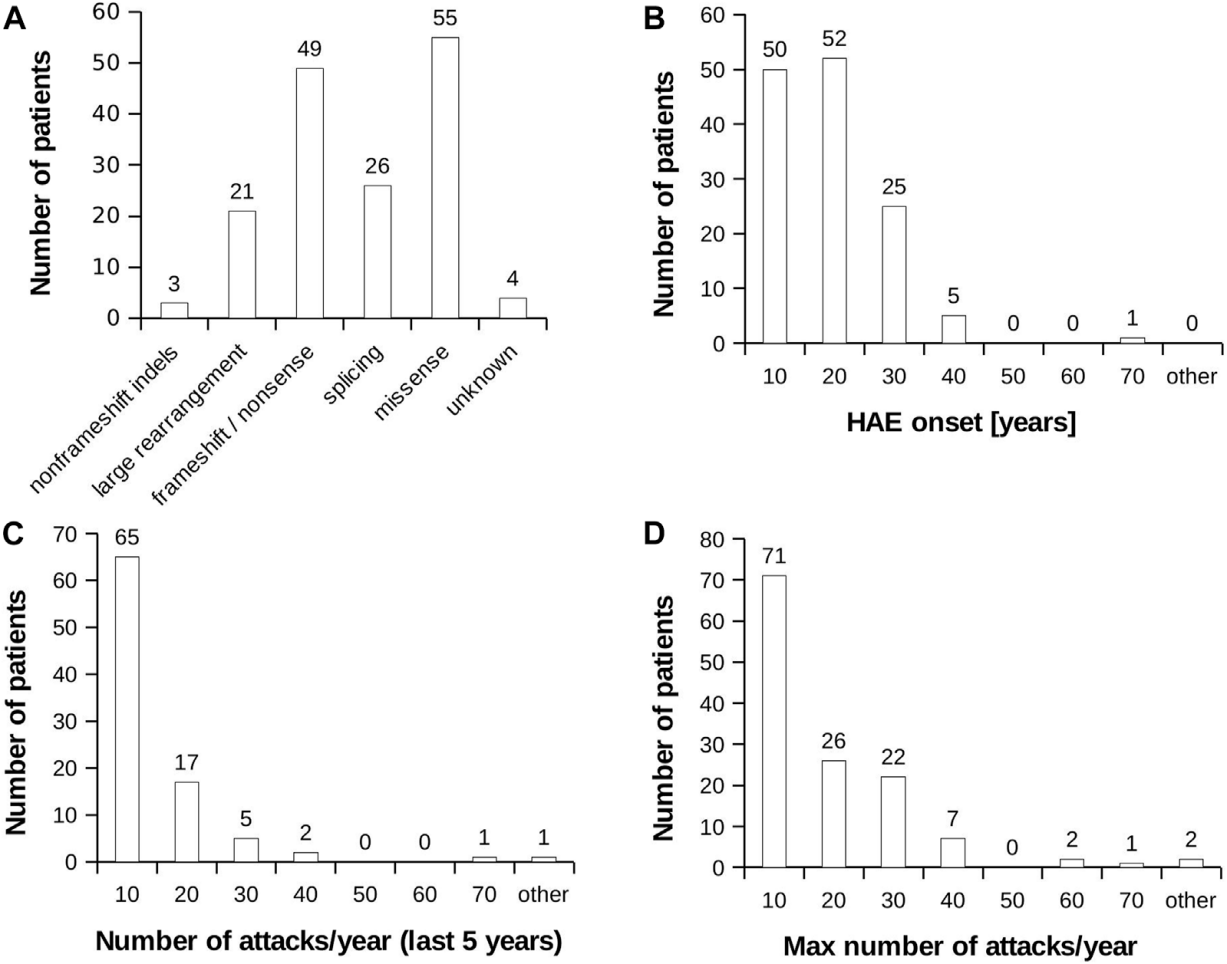
HAE patient characteristics. **(A)** Distribution of mutation type in the analysed HAE patient cohort. **(B)** Histogram representing age at HAE onset. **(C)** Histogram representing average number of recorded attacks per year. The value was counted from last 5 years. **(D)** Histogram representing highest recorded number of attacks per year during the whole monitored period of patient’s life.

None of selected attributes distinguish patients into naturally separated groups and the values corresponded to right-skewed distribution (Figure 1). Therefore, we defined severe and mild subgroups according to arbitrarily defined values with respect to known HAE characteristics. Thus, classification criterion (CC) CC1 reflected an age at disease onset around puberty as an important life period, when the number of attacks grows (Sabharwal and Craig, 2017). CC1-M thus corresponds to the period after the latest age and CC1-S before the earliest age of puberty. Otherwise, we distinguish long-term, reflecting lifelong disease severity [the average number of attacks per year during the last 5 years (CC2) and the all-time maximum number of attacks per year (CC3)] from short-term, reflecting patients’ status close to the time of blood collection [the number of attacks in the last year before sample collection (CC4)] (Table 1; Supplementary Table S1). The main threshold in CC4 was assessed as 5 per year, determined based on the attack number distribution reflecting a median in our patient cohort. As the patient cohort for DNA analysis contained a higher number of patients, the median number of attacks shifted a little bit in long-term criteria CC2 and CC3 (Table 1). The thresholds were established to exclude patients with attack numbers around this threshold (plus minus one decile). This limitation should prevent patients with temporarily affected values being assigned to the incorrect subgroup. The distribution of individual characteristics in particular CC categories is summarized in Supplementary Figure S1.

**TABLE 1:** Classification of HAE patients to severity subgroups. CC groups do not contain the same patients and therefore, depending on the criteria used, there is only a partial overlap. Group CC5 contains only patients included in CC4. CCx-S = severe HAE course, CCx-M = mild HAE course; CC5-Y = on long-term prophylaxis (LTP), CC5-N = without LTP. Details about LTP are summarized in Supplementary Figure S1.

Classification criterion (CC)	CC subgroup	Number of analyzed patients	Applied threshold for a CC subgroup	Median	Minimal value	Maximal value
**CC1 (age of HAE onset)**	CC1-S	50	≤11 years	6	1	11
	CC1-M	55	>15 years	20	16	68 (+3 patients asymptomatic)
**CC2 (attack number—average in last 5 years)**	CC2-S	36	≥6 attacks/year	13.4	6	75.6
	CC2-M	34	<3 attacks/year	0.6	0	2.8
**CC3 (attack number—max in last 5 years)**	CC3-S	51	>13 attacks/year	24	14	81
	CC3-M	55	<6 attacks/year	2	0	5
**CC4 (attack number in last year)**	CC4-S	20	≥5 attacks/year	12	5	72
	CC4-M	20	<5 attacks/year	0	0	4
	controls	20	NA	NA	NA	NA
**CC5 (long-term prophylaxis)**	CC5-Y	13	treated	NA	NA	NA
	CC5-N	27	non-treated	NA	NA	NA

If a genetic predisposition to a more serious lifelong disease manifestation is analysed, applying permanent or long-term criteria (CC1-CC3) seems to be more relevant as permanent gene variants act during the patient’s whole life. On the other hand, the current homeostasis and gene expression profile is more related to short-term disease characteristics as defined by CC4. Therefore, we applied CC1—CC3 to assess gene variants on HAE severity and CC4 for monocyte/macrophage transcriptomic analysis. Despite this sorting, patients’ distribution to CC2 and CC4 subgroups correlated well, just that some patients were assessed as severe or mild in CC4, while in CC2 as intermediate (Supplementary Table S1).

### 2.2 Genetic background in HAE-C1INH patients

To uncover gene variants potentially involved in a diverging effect of C1 inhibitor insufficiency, we searched for DNA variants in selected genes using DNA-seq. The sequenced genes (181 in total) were chosen based on their confirmed or hypothetical involvement in HAE. Generally, they could be divided into genes involved in bradykinin metabolism and signaling, regulation of endothelial barrier function, monocyte/macrophage differentiation and activation, and inflammatory responses (Supplementary Table S2).

#### 2.2.1 Detection of rare deleterious variants in HAE patients

To inspect the presence of potentially deleterious gene variants, all identified variants were restricted to exonic variants substantially changing the amino acid composition (nonsense, or frameshift variants), or to single amino-acid substitutions with a predicted high probability of deleteriousness (CADD score higher than 18; and 2 of 3 predictors - PolyphenHVAR, PolyphenHDIV, SWIFT - with deleterious predictions) and with a population frequency lower than 0.05 (according to the Genome Aggregation Database). The acquired list contained variants including those localized in the *FXII, ANGPT1* or *KNG1*—genes previously published as being associated with nC1-INH HAE development. Applying CC1-CC3 did not reveal any significant change in the number of such variants between severe and mild subgroups (data not shown). Most such variants (6) were detected in the *thrombospondin 1 (THBS1)* gene, while their maximal number in other genes did not exceed three (Supplementary Tables S3, S4). However, their contribution to HAE development remained unclear. Therefore, we tested these variants’ segregation (incl. those found in *IL6ST, THBS1* or *IRAK2*) with the HAE severity in larger families. No single variant segregated consistently with either a mild or severe HAE phenotype.

#### 2.2.2 Association of common SNPs with HAE phenotype

Additionally, we tested whether gene variant frequencies were altered in patients with severe versus mild HAE. Chi-square statistics with Bonferroni correction of the *p*-value was applied to prove, whether the variant frequency in any subgroup (CC1-3 S/M) is under/over-represented. As the population frequency in our Czech patient cohort differed significantly from database values (gnomAD, 1000 g) in many cases, the variant frequencies calculated from all sequenced individuals were used as expected values. Considering a relatively low prevalence of asymptomatic HAE carriers (Aygören-Pürsün et al., 2018), this frequency should correspond to overall frequencies in the Czech Republic and a statistical test should thus determine the variants as significantly different only if their frequencies are under/over-represented in the appropriate subgroup at the expense of the rest of the individuals tested. None of the identified variants exceeded the given *p*-value (Supplementary Table S5), which suggests, that the severity defined by our CC was not determined by any of these gene variants.

### 2.3 Transcript profiling of monocytes and macrophages

**FIGURE 2:**
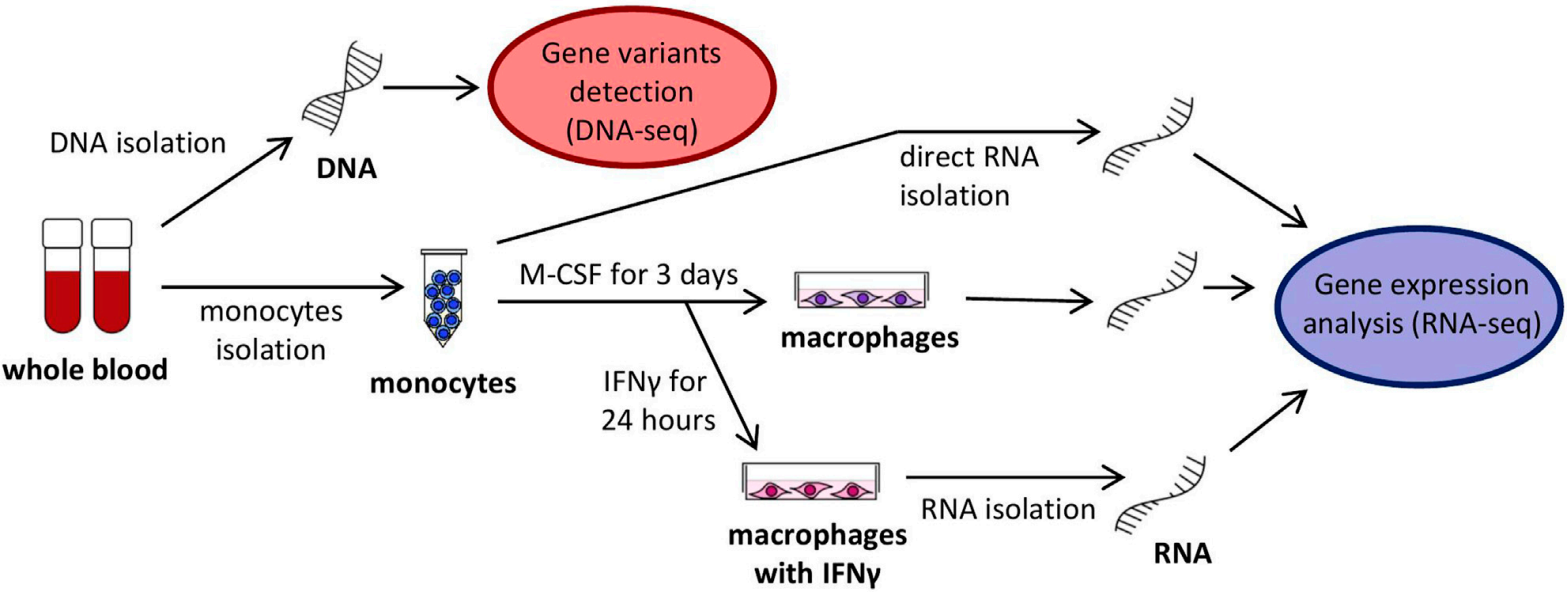
Scheme of analyzed cells, their cultivation and harvesting.

Peripheral monocytes were isolated from the blood in a symptom-free period and stimulated to differentiate to macrophages M0 and subsequently activated by interferon-gamma (IFNγ) to induce differentiation to macrophages M1 (Figure 2). It has previously been demonstrated that IFNγ application leads to an elevated *SERPING1* mRNA level not only in hepatocytes (Zuraw and Lotz, 1990), but also in monocytes (Lappin et al., 1989), which could be a supplementary source of C1 inhibitor (Lappin et al., 1992). It can be hypothesized that mRNA steady state levels of some genes, including *SERPING1*, could influence monocyte activity, and also HAE pathogenesis and disease course indirectly. Therefore, the expression pattern of the chosen candidate genes was assessed using targeted RNA-seq in all of these cell types. The analysed genes (141 in total, incl. potential housekeeping genes) were selected based on their expression in monocytes/macrophages and their involvement in HAE or monocyte function (Supplementary Table S6). RNA-seq data was validated using quantitative PCR for *CD74* and *CXCL10* in monocytes, *CCL2* and *CXCL8* in macrophages and *SERPING1, NEAT1* and *ACE* in macrophages treated with IFNγ (Supplementary Figure S2). They were selected to cover up-, down- or non-regulated genes. Although the absolute mRNA steady state level fold changes were slightly different in both methods, the overall trend remained the same, which indicated sufficient RNA-seq reliability.

#### 2.3.1 Differential transcript profiles in HAE patients’ myeloid cells

**FIGURE 3:**
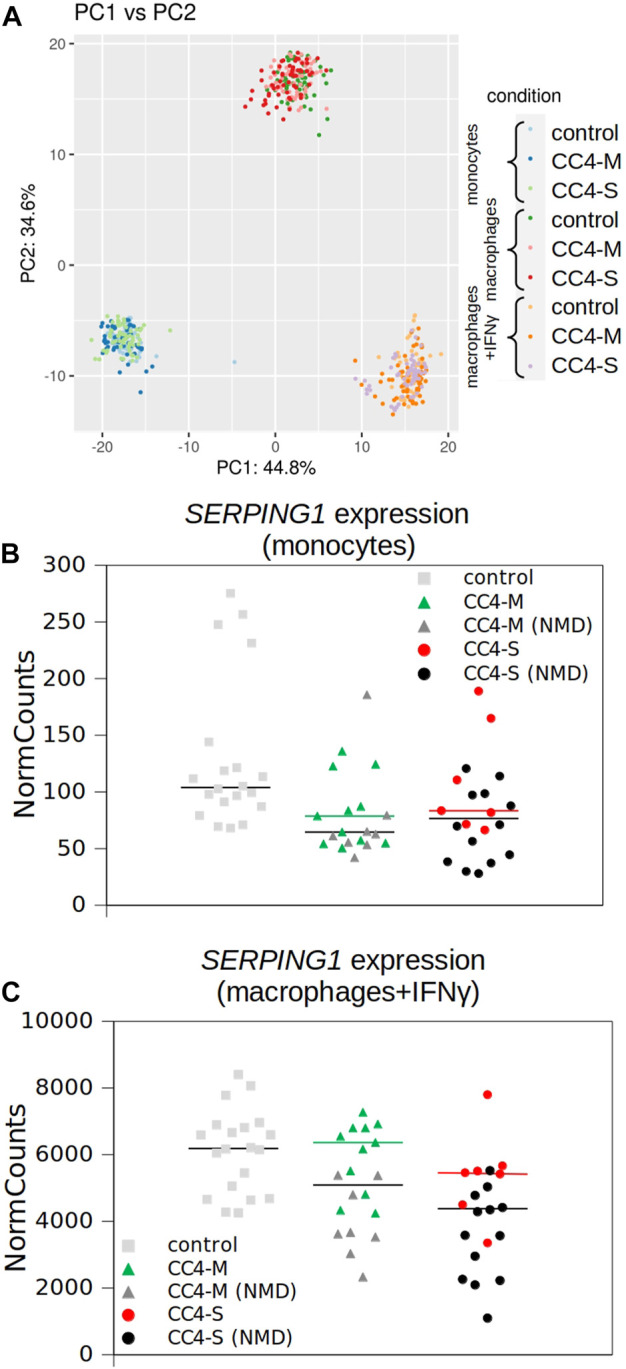
Transcriptional profiling of monocytes and macrophages by RNA-seq. **(A)** Similarity of whole transcript profiles acquired by principal component analysis (PCA). Individual samples were preferentially clustered by cell type and not by patient categorization and any patient’s subgroup was not separated from others. PCA of expression data (normalized using variance stabilizing transformation (VST) in Deseq2) was conducted in R using the prcomp function and visualized using ggplot2 library. Variances explained by PC1 and PC2 are shown in %. Depicted points represent analysed samples including the corresponding biological replicates. **(B, C)** SERPING1 expression extracted from RNA-seq data. Normalized read counts adopted from Deseq2 outputs and visualized for monocytes and macrophages treated with IFNγ independently. Horizontal black bars represent medians for all patients and colored for patients carrying SERPING1 mutations, which should not induce RNA degradation by NMD. Corresponding SERPING1 mRNA levels for particular patients are shown in the same color.

Overall differences in gene mRNA expression patterns between controls and HAE patients with mild and severe disease courses were lower than between cell types, as no HAE/control subgroup formed an isolated cluster in principal component analysis (PCA; Figure 3A) and samples derived from patients’ cells were indistinguishable from control samples. Although the differences in the overall expression pattern were very small between groups of patients/controls, individual genes differing in particular transcript levels were identified.

**FIGURE 4:**
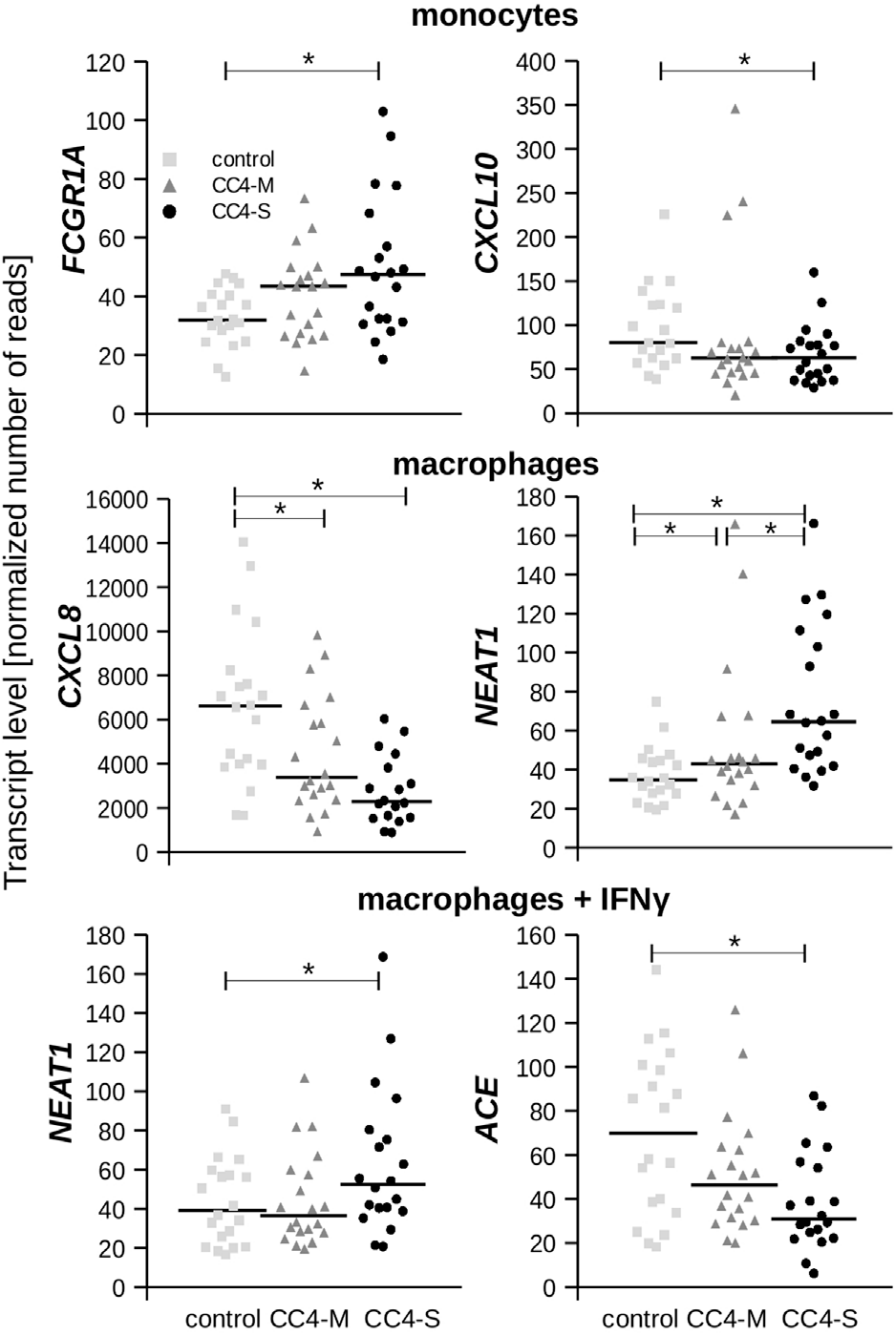
Transcript levels of chosen genes. Plots depict normalized read counts adopted from Deseq2 program for controls and HAE patients categorized to CC4-M and CC4-S. Horizontal bars represent median. Statistical significance is depicted as a star (*p* < 3.65E-04).

While a significantly different mRNA level was detected between controls and HAE patients in some genes (either CC4-M or CC4-S, or both), similar changes between CC4-M and CC4-S patients were considerably rarer (Figure 4; Table 2; Supplementary Table S6). In total, four genes in monocytes, five in macrophages and four in macrophages treated with IFNγ were up/downregulated in at least one patient group compared to controls. On the contrary only *NEAT1* and *IL1RN* were upregulated in CC4-S compared to CC4-M in macrophages and *NEAT1* and *CCL3L3* in macrophages treated with IFNγ. Also, SERPING1 gene was identified among the genes downregulated in HAE patients’ monocytes and macrophages treated with IFNγ, while not in untreated macrophages, as its steady state mRNA level was too low when compared to other cell types. Lower *SERPING1* mRNA levels in HAE patients compared to controls supported the reliability of acquired data, and, further, it could be a reason for HAE severity diversification. However, this latter hypothesis was not proved in our datasets, as the difference in *SERPING1* transcript level was not significantly different between CC4-S and CC4-M. The transcript level was related to the *SERPING1* variant type rather than HAE severity, as the carriers of *SERPING1* variants susceptible to nonsense-mediated decay (NMD) degradation had a lower transcript level than those with other variant types and were similarly represented in both CC4-M and CC4-S groups (Figure 3).

**TABLE 2:** Differentially expressed genes in HAE patients’ monocytes/macrophages. Genes evaluated as differentially expressed between groups showed statistically significant values compared to the control group. The arrow indicates a stronger effect (log2fold change) in patients with severe HAE, although the difference between CC4-S and CC4-M is not significant. Contrarily, genes in bold showed significantly different expression between CC4-S and CC4-M. *For *IL1RN*, upregulation was observed only comparing CC4-S with CC4-M, and not with the control group. *SERPING1* gene was left out as its mRNA level can be posttranscriptionally degraded in patient cells by NMD.

	Monocytes		Macrophages		Macrophages + IFNγ	
	CC4-M	CC4-S	CC4-M	CC4-S	CC4-M	CC4-S
Upregulated genes	*—*	*FCGR1A*	***NEAT1***	*NEAT1*	*—*	***NEAT1***
			*CD180*	IL1RN*		
Downregulated genes	*—*	*CXCL11*	*CXCL8*	*CXCL8↓*	***CCL3L3***	*ACE*
		*CXCL10*	*CLEC4E*	*CLEC4E*		
				*IL1B*		

From the genes encoding bradykinin-metabolizing enzymes, only *ACE* was downregulated in macrophages treated with IFNγ. On the contrary, chemokines prevailed among genes with decreased expression in both HAE patient groups. Although the changes in mRNA levels reached statistical significance in only one cell type, the same trend could also be detected in other cell types, as can be demonstrated in *CXCL10* and *11*, in which reduced expression was also observed in macrophages, but not in those treated with IFNγ. In fact, IFNγ is one of the most potent activators of *CXCL10* and *11* expression (Neville et al., 1997; Mazanet et al., 2000), which apparently surpassed the effect accompanying HAE in our experiment.

To rule out the possibility that long-term prophylaxis (LTP) could influence a transcript profiles, we implemented an additional classification criterion CC5 considering this type of treatment. CC4-M and CC4-S included 6 and 7 patients, respectively, receiving LTP (CC5-Y) (Supplementary Table S1). Comparing CC5-Y and CC5-N subgroups revealed no significant changes in transcript levels between them in monocytes and macrophages and only slight *IL1B* upregulation in M1 macrophages. This data suggests a low dependence of obtained transcript profiles on HAE treatment and rather corresponds to changes accompanying HAE *per se* (Supplementary Table S2, Supplementary Figure S3). However, it should be stressed, that different types of LTP were used, which could mask the effect of individual drugs on transcript profiles.

**FIGURE 5:**
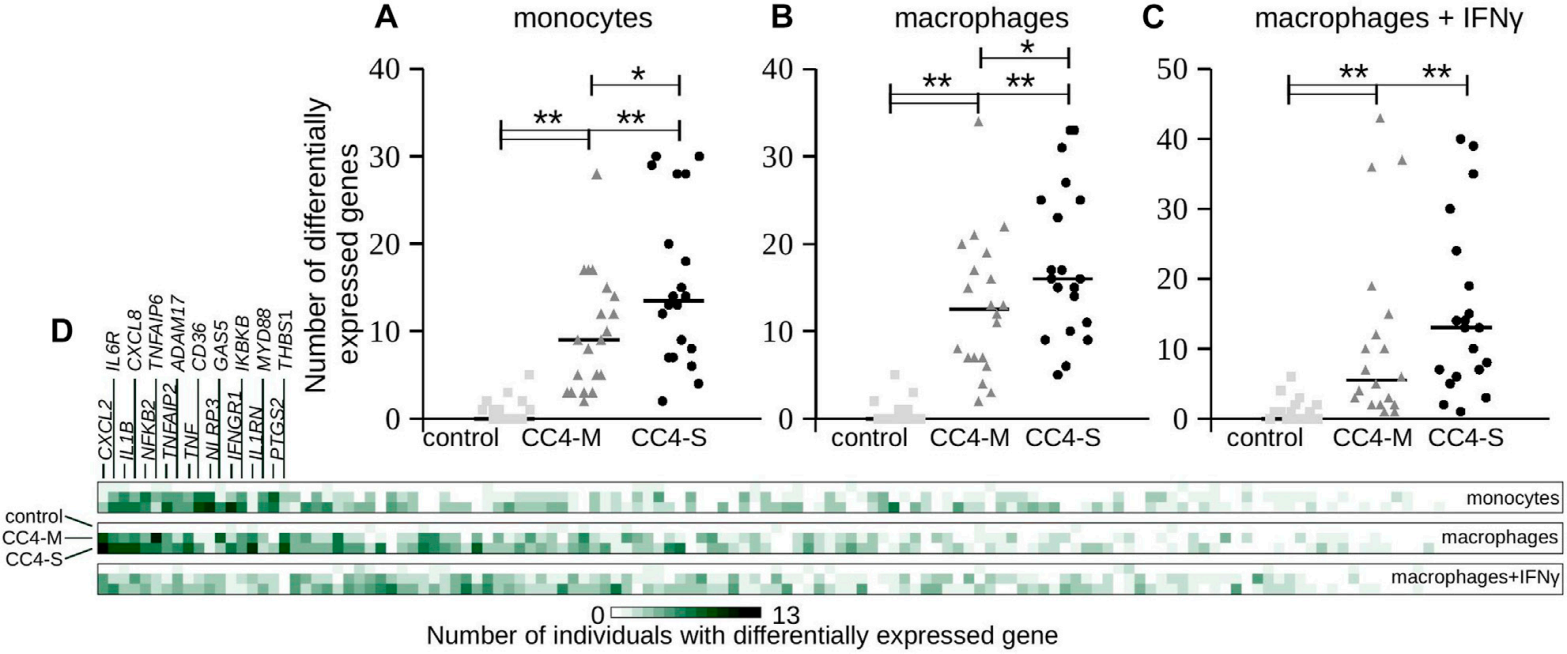
Transcript levels at individual level. **(A–C)** Each gene transcript level of all analyzed individuals in the study was compared with the transcript level defined by all members of the control group in CC4. Plots depict absolute number of genes having a statistically different (positively or negatively) transcript level (*p* < 3.65E-04) compared to the control group of CC4 for each individual separately. Each dot thus represents one individual and horizontal bars median counted for the whole group. Differences between groups were evaluated by Mann-Whitney test. *p < 0.05. **p < 0.01. **(D)** Heat map representing the same analysis from a gene perspective. Color intensity corresponds to the absolute number of individuals having statistically different transcript levels for each gene. First 18 genes are enumerated.

Considering that HAE severity can be modulated by individual predispositions, differences in individual gene expression were checked by comparing every single HAE patient with the control group regardless of positive or negative shift in relation to this control. In all cell types, both CC4-S and CC4-M patients displayed a higher number of genes, whose transcript levels were outside of the interval defined by control group for CC4. Additionally, CC4-S patients displayed a higher number of these genes than CC4-M in all studied cell types (Figure 5). This effect was lower for macrophages treated with IFNγ. Genes identified by this approach did not fit completely with those identified by comparing whole groups, which was the result of higher interindividual variability in CC4-M or -S than in healthy controls. Interestingly, such dysregulation was specific only for a low number of analysed genes, while the others retained expression levels within the limits observed in the control group (Figure 5D). Based on this specificity, we speculated that the state of the transcriptional regulatory network in the studied cells was not imbalanced (in the sense of randomness), but rather shifted to a different stable or metastable state.

#### 2.3.2 Specific gene co-expression in myeloid cells

**FIGURE 6:**
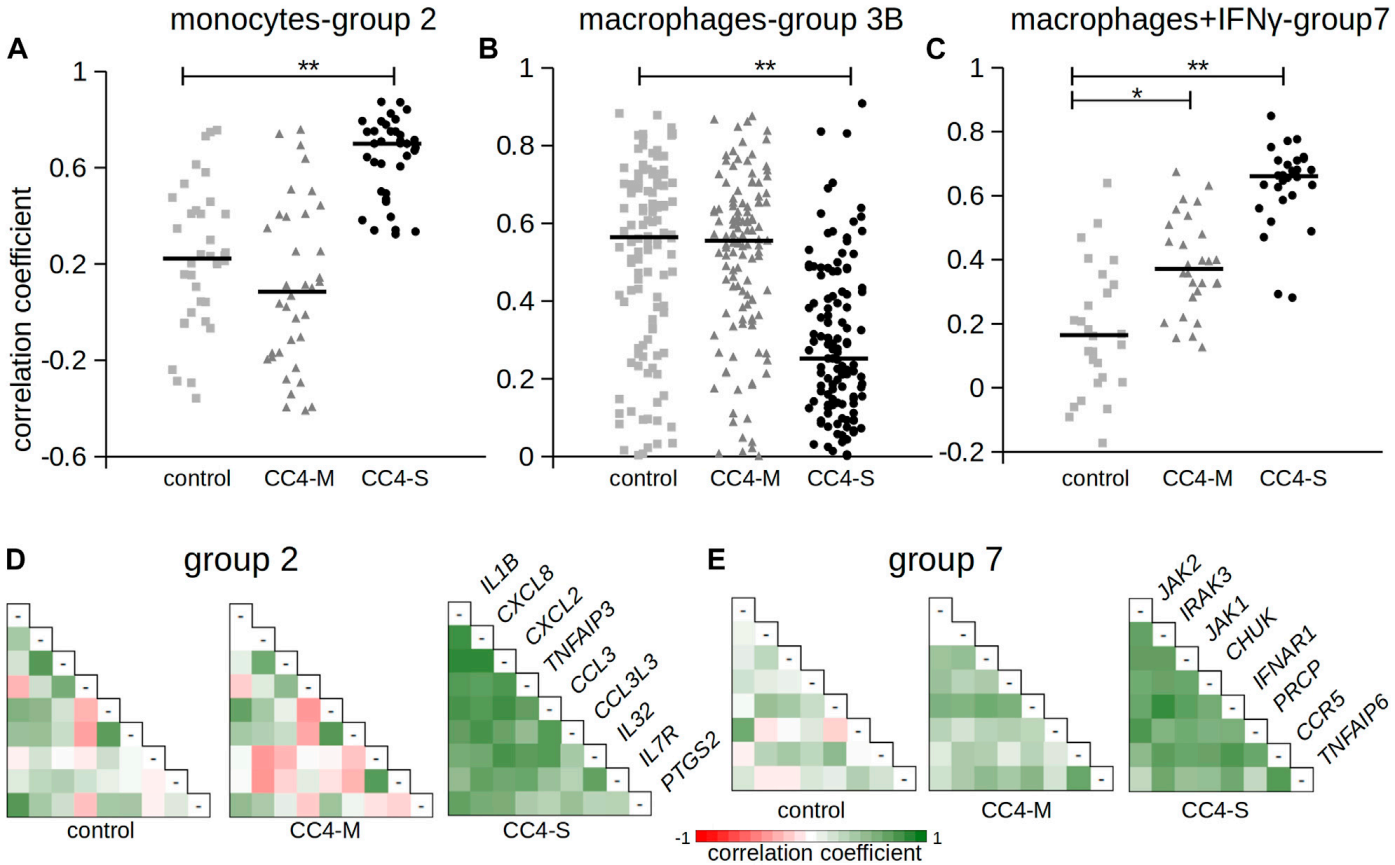
Correlation of gene expression between chosen genes. The figure shows Pearson correlation coefficients in selected gene groups. Complete results are summarized in Table 4 and Supplementary Table S7. **(A–C)** Pearson correlation coefficients calculated for gene groups 2, 3B and 7. Horizontal bars represent median. Differences between groups evaluated by Wilcoxon test. *p < 1.00E-03, **p < 1.00E-07. **(D, E)** Pearson correlation coefficients depicted as a heat map. Gene names refer to the corresponding row and column in combinatorial table.

To test if monocyte/macrophage status can be shifted toward a different transcriptional state in dependence on HAE severity, we calculated the correlation coefficients for all gene combinations and searched for the groups of co-expressed genes in CC4-M, -S or controls independently. The groups were defined in the patient’s subgroup with the highest correlation coefficients (exceeding 0.7 in at least one comparison) for a particular cell type (Table 3; Supplementary Table S7). Most of the genes thus identified belonged to one predominant group (group 1 in monocytes, 3 in macrophages and 6 in macrophages treated with IFNγ). These groups contained members with positively (subgroups A and B) and negatively (correlation between A and B) correlated mRNA levels. Comparing these gene groups showed significant differences between CC4-M/S and the control and even between CC4-M and CC4-S (Figure 6; Table 4).

**TABLE 3:** Identified groups of coregulated genes in monocytes and macrophages. Each group represents genes with interconnected expression in at least one patient/control group and one cell type (according to the correlation coefficients). Groups were formed with respect to cell type. Thus, particular genes can be found in more groups. Groups 1, 3 and 6 were divided into A and B subgroups. Correlation coefficients were positive inside these subparts and negative between them.

Cells	Group	Genes
monocytes	1A	*TLR4, PRCP, IFNAR1, IFNGR1, CD36, C1QBP, HSP90AB1, HSP90AA1, IMMT, IFNGR2, CASP1, IL1RN, MYD88, CPM, TRAF6, TNF, CXCR2*
	1B	*TRAF1, REL, IKBKB, NEAT1, NLRP3, RBM15, IRAK3, SEMA4D, XPNPEP1, KIAA0586, NFKB2, GBP2, TNFAIP2, ARRB2, ZBTB22*
	2	*IL1B, CXCL8, CXCL2, TNFAIP3, CCL3, CCL3L3, IL32, IL7R, PTGS2*
macrophages	3A	*CD180, GPBAR1, TRAF6, IL10, CD14, CD36, TNFSF13B, TCEAL1, DCAF10, TLR4, CPM, CASP4, IRAK4, CASP1*
	3B	*SERPINE1, NFKB2, TRAF2, TRAF1, ACE, TNFAIP2, IL7R, TNFSF14, HSP90AB1, ITGAM, CSF1, CCL24, CD274, IL17RA, MMP9, IKBKB*
	4	*CXCL8, CCL3, NCF1, IL1B, CXCL2, TNFAIP3, CCL3L3, CXCL1, TNFAIP6, TNF, CLEC4E*
	5	*IL1RN, PLAUR, TIMP3, TNFAIP8L3, MMP7, IL1R1*
macrophages + IFNγ	6A	*IL10, TNFSF13B, CASP1, SOCS1, IFNGR2, CXCL10, C1QBP, CD180, CASP4, TNFRSF1A, CD36, MYD88, CD14, SERPINB9, IFNGR1, MAFB, STAT1, TLR4, CXCL11*
	6B	*PLAUR, REL, IL1RN, TRAF3IP2, TNFSF14, TIMP3, IL6R, EPAS1, ADAM17, SERPINE1, CSF1, CCL3L3, CCL3, TRAF1, TNFAIP8L3, CXCL8, ARRB2, IRAK2, MMP7, CPM, IL17RA, VEGFA, ITGAM, CD274*
	7	*JAK2, IRAK3, JAK1, CHUK, IFNAR1, PRCP, CCR5, TNFAIP6*

**TABLE 4:** Correlation rate inside gene groups. Table contains medians of all unique correlation coefficients counted inside the gene subgroup. Negative correlations were only compared for appropriate subgroups (A-B). The values in bold are statistically different (Wilcoxon test; *p* < 0.01) compared with controls (healthy individuals).

Gene group		Monocytes				Macrophages					Macrophages + IFNγ			
		1A	1A-B	1B	2	3A	3A-B	3B	4	5	6A	6A-B	6B	7
Pears correl coef	control	0.57	−0.39	0.39	0.22	0.4	−0.45	0.56	0.31	0.52	0.54	−0.56	0.61	0.16
	CC4-M	0.55	−0.42	**0.69**	0.08	**0.56**	**−0.5**	0.56	**0.56**	0.39	0.53	−0.55	0.64	**0.37**
	CC4-S	**0.47**	−0.41	**0.55**	**0.7**	0.31	**−0.19**	**0.18**	**0.49**	0.6	0.51	**−0.45**	**0.5**	**0.66**

In monocytes, genes belonging to groups 1B and 2 were more tightly co-expressed, especially in CC4-S. A targeted, candidate gene-based approach to the RNA-seq experiment did not allow us to use statistical tests for functional characterization of these groups. However, simplified analysis (using Gene Ontology Resource; Ashburner et al., 2000) revealed group 1B enrichment with genes involved in NF-kappaB function (*IRAK3, IKBKB, TRAF1, NLRP3, ARRB2, NFKB2*), while group 2 remarkably involved gene encoding cytokines. In macrophages, especially CC4-S patients lost the majority of interconnections in the group 3B, while group 4 displayed a higher average correlation coefficient in both CC4-S and CC4-M. Group 4, containing more cytokines including *CXCL8, IL1B, CXCL1*, and *TNF*, was strikingly similar to group 2, and seemed to also preserve the same trend when the CC4 subgroups were compared. The most striking effect was observed in macrophages treated with IFNγ, where the correlation in the group 7 rose sharply in the order of control < CC4-M < CC4-S (Figure 6; Table 4).

## 3 Discussion

This study aimed to characterize modifying factors explaining variable expressivity of hereditary angioedema (HAE-C1INH). This phenomenon seems not to be directly dependent on the type of *SERPING1* mutation as the carriers of the same pathogenic *SERPING1* variant were shown to exhibit a highly variable phenotype ranging from asymptomatic disease course up to frequent occurrence of life-threatening attacks (Rijavec et al., 2013; Hujová et al., 2020; Parsopoulou et al., 2022), making it apparent that other genetic and/or environmental factors have an impact on the clinical phenotype. Thus searching for these HAE modifying factors is a challenging mission. In last years, several approaches were applied to deal it. These approaches included linkage/association studies with known genes potentially affecting HAE severity (Freiberger et al., 2011; Gianni et al., 2017; Parsopoulou et al., 2022), screening of the larger sets of such genes using NGS analysis (Veronez et al., 2019), RNA-based studies analysing the whole transcriptome of PBMC (López-Lera et a., 2013; Castellano et al., 2018) or analysing individual gene expression changes in different cells (Grymová et al., 2019; Farkas et al., 2022).

From the perspective of prophylactic therapies, knowledge of factors predisposing attack occurrence can help clinicians fine-tune HAE management and improve the patient’s quality of life at the individual level. However, HAE severity is difficult to exactly characterize for several reasons. Disease manifestation is highly variable over time, and can occur with diverse intensity, which might be interpreted differently by patient and clinician. Laryngeal attacks are assessed as more severe than others, although it is not clear if the severity (in terms of genetic predisposition) is correlated with the swelling location, and last but not least the applied therapy hinders correct disease severity quantitation (Bygum et al., 2017). To avoid questionable approaches to scoring HAE severity, we have simplified the categorizing criteria to unambiguously definable categories derived from the age of HAE onset and the number of attacks. As the current state of the organism could affect the patient’s susceptibility to an attack occurring, we have distinguished long-term criteria from short-term. While applying long-term criteria seems to be more relevant for studying genomic variants modifying HAE severity, short-term criteria were applied for the transcriptomic study.

### 3.1 Identification of HAE severity modifiers in the genome

Using targeted DNA-seq, we searched for gene variants suggested to be involved in HAE pathogenesis. We identified several potentially deleterious variants that had not been previously identified, or whose population frequency was very low. The significance of most of them was hardly interpretable, because they were found only in one patient and only a few of them were present in more family members. However, no gene variant was associated with HAE severity in these families. As both severe and mild HAE phenotypes are relatively abundant, the modifying alleles should not be rare. We searched for alleles up- or down-represented in HAE subgroups sorted based on disease severity with a negative outcome. Moreover, some alleles previously described as associated with HAE severity were included in both -S and -M subgroups in a similar frequency. Thus, a c.-4T>C variant in FXII (rs1801020) was not over-represented in any HAE subgroup, while Corvillo et al. observed this FXII polymorphism in association with the HAE-FXII severity score (Corvillo et al., 2020), Bors et al. with the age of HAE-I onset (Bors et al., 2013) and Parsopoulou et al. (2022) with both age at HAE onset and Cutaneous Abdominal Laryngeal severity Score (CALS) of HAE-I and -II patients. Similarly, no association was confirmed for c.428G>A *KLKB1* (rs3733402) (Gianni et al., 2017), c.533G>A *CPN1* (rs61751507) (Parsopoulou et al., 2022) or *SERPING1* variants (Supplementary Table S5). These associations were not confirmed by applying the same CCs as in the cited works either (Supplementary Figure S4). All these data indicate that the modifying effect is not linked to our sequenced loci, or that no identified single gene variant is a dominant factor and multiple variants/factors must be combined.

One of the anticipated hypotheses supposes that the current C1 inhibitor level is a dominant driver of HAE severity. This assumption is in good agreement with the observed relationship between functional C1-INH levels and attack occurrence (Suffritti et al., 2014), which can be efficiently treated by subcutaneous C1 inhibitor administration (Kaplan et al., 2020). The association between HAE severity and C1 inhibitor level has been shown before (Kelemen et al., 2010), but the nature of this phenomenon has not been explained completely. HAE is an autosomal dominant disease and patients are heterozygous for the defected *SERPING1* allele. However, C1 inhibitor’s functional level is sometimes below 50%, sometimes reaching very low or undetectable levels (Cicardi et al., 1987; Honda et al., 2021), which can be interpreted as reduced production from both alleles in these specific cases (Kramer et al., 1993). Haslund et al. (2019) showed that the dominant-negative effect of mutant *SERPING1* alleles can inhibit the secretion of wild-type C1 inhibitor from hepatocyte-like cells by forming intracellular C1 inhibitor protein aggregates. Besides, the mRNA level itself could also stand behind this effect. However, some contradictory data have been gathered in previous years. While Pappalardo et al. documented a decreased mRNA level regardless of the *SERPING1* mutation type (Pappalardo et al., 2004), this dependence was not confirmed in a family-based study (Kang et al., 2006), or in another larger cohort study (de la Cruz et al., 2012), where missense mutations did not lead to significantly lower mRNA levels. In our study, the overall *SERPING1* mRNA level was lower in both HAE patient groups, even more apparently in the severe HAE subgroup when stimulated by IFNγ. However, this association disappeared when patients carrying mutations causing premature stop codon incorporation (such as splicing, nonsense, or frameshift mutations) and subsequent mRNA degradation by NMD were excluded from the analysis, which indicated that NMD was a predominant factor responsible for this decrease. Applying the assumption that the expression in monocytes corresponds to the expression in hepatocytes (as the main source of the plasma C1 inhibitor), it can be concluded that lower *SERPING1* mRNA level caused by a factor other than NMD is not responsible for a diverse HAE phenotype, or that this effect is relatively low compared to the predominant NMD influence.

### 3.2 Monocytes in HAE course

Transcriptomic analysis performed in monocytes and two types of macrophages revealed changed transcript levels in all cell types of several genes in HAE patients compared to healthy controls. On the contrary, the differences between two groups of patients were less significant, although a trend in discriminating between HAE patients with mild and severe disease course was apparent based on transcript levels of some genes.

In monocytes, *FCGR1A* was upregulated and *CXCL10* and *CXCL11* downregulated with a stronger effect in patients with severe HAE. Both CXCL10 and 11 are pro-inflammatory cytokines positively regulated by IFNγ (Hanaoka et al., 2003) and their increased activity is associated with different immune disorders (Lee et al., 2009). A diverse mechanism can impact these cytokines’ basal expression by monocytes and the potential influence of IFNγ endogenous level or decreased sensitivity to IFNγ cannot be excluded. We measured IFNγ level in blood plasma to verify if IFNγ should be such an effector, but the IFNγ levels neither differed between HAE patients and controls, nor correlated with the *SERPING1, CXCL10* or *CXCL11* mRNA levels (data not shown).

Contradictorily, *FCGR1A* showed the opposite effect. *FCGR1A* encodes Fcγ receptor I (FcγRI), which is a high affinity receptor for immunoglobulin G (IgG) expressed on the surface of monocytes. FcγRI expression is correlated with immune inflammation and supports inflammasome formation and IL-1β release (Zhang et al., 2018). Its overexpression leads to enhanced IL-1β and NLRP3 expression. Such activation was not observed in our patient cohorts. We suggested that this effect was dose-dependent and did not reach a sufficient intensity to regulate a cell response at the transcriptional level. However, FcγRI also seems to be a potent NF-κB pathway activator after binding IgGs (Kecse-Nagy et al., 2016) and NF-κB contributes to the differentiation of monocytes into macrophages (Takashiba et al., 1999). Correlation analysis in our study revealed that genes in group 1B were more tightly co-expressed in HAE patients than in controls, and group 1B included a lot of genes involved in the NF-κB pathway, although their mRNA level was not affected. The NF-κB pathway thus can play a role in HAE pathogenesis. NF-κB activation within endothelial cells contributes to the increased endothelium permeability (Sehnert et al., 2013; Terzuoli et al., 2014) and secondly, bradykinin can induce NF-κB activation through its BDKRB2 receptor (Chen et al., 2004) in epithelial cells.

There is a question whether monocytes and macrophages can also be affected and whether individual differences in these pathways can explain diverse HAE phenotypes. Anyhow, bradykinin can also act through NF-κB signalling pathway in monocytes (Dong et al., 2015) by inhibiting tissue factor expression through bradykinin receptor BDKRB2 (Bourdet et al., 2010). Strengthened co-expression of subgroup 1B genes especially in patients with mild HAE (CC4-M) indicates that NF-κB signalling pathway might be alerted and brought to a specific inhibitory regulatory state in these patients. Thus, synergic action of NF-κB signalling pathway members in monocytes of genetically predisposed HAE patients could prevent them from the higher number of attacks and make their disease course milder. Supporting arguments for this hypothesis can be found in Papoutsopoulou et al. (2019), where the authors observed that inducing the NF-κB pathway in macrophages displayed sufficient variability to enable division of inflammatory bowel disease (IBD) patients into different clusters. On the other hand, correlation coefficients in CC4-S patients were lower than CC4-M, but still higher than in controls, suggesting another possible explanation. Tightened transcript levels could be a post-regulatory consequence of HAE attacks or other physiological processes preceding blood collection. Monocytes isolated from peripheral blood thus could reflect a history occurring in patients’ blood.

Long-term prophylaxis could be, why immune cells exhibited decreased steady-state level of transcripts associated with inflammatory responses. Indeed the drugs used, such as danazol or berotralstat, could induce an anti-inflammatory state in the studied cells. However, in our experiments, no difference between on-LTP (CC5-Y) and without LTP (CC5-N) patients was shown, and the distribution of CC5-Y samples seemed independent on HAE severity in transcriptomic data (Supplementary Figure S3). The CC5-Y group included patients with different types of treatment (danazol, berotralstat, tranexamic acid, or their combinations) acting by different mechanisms and, thus, likely affecting monocytes differentially. It may be the reason, why we did not observe any significant difference both between the CC5-Y and CC5-N and between representation of patients on-LTP in the CC4-S and CC4-M subgroups. Despite the anti-inflammatory capacity of the drugs used, the measured transcript levels were probably affected by factors other than the applied treatment.

Concerning the observed effects in macrophages M0 and M1, we considered if the results do not reflect artificial *ex vivo* cultivation conditions rather than patients’ cell properties. However, statistically significant differences observed between controls and HAE patient subgroups (Supplementary Table S7, correlation group 7) argue in favour of the latter option.

### 3.3 Macrophages in HAE course

In macrophages, *IL1B* and *CXCL8* were downregulated in HAE patients depending on disease severity and *CLEC4E* in both HAE groups comparably. *NEAT1* and *CD180* were upregulated and surprisingly *IL1RN* was also upregulated in patients with a severe phenotype compared to patients with a mild one. In our previous work (Grymová et al., 2019), we showed that *CXCL8* expression was higher in HAE patients’ neutrophils, while its plasma level was decreased. This discrepancy is now easily interpretable if we consider different CXCL8 resources in the plasma. CXCL8 serves as a chemotactic molecule for monocytes and neutrophils (Zhang and Chen, 2002) and plays an important role in inflammatory diseases such as rheumatoid arthritis, IBD or psoriasis (Seitz et al., 1991; Schröder et al., 1992; Grimm et al., 1996). In HAE patients, CXCL8 level was only increased during attacks (Veszeli et al., 2015; Kajdácsi et al., 2021), but not in asymptomatic periods (Demirtürk et al., 2014; Grymová et al., 2019), which is consistent with non-inflammatory macrophages’ state between attacks. Decreased expression of inflammatory *CXCL8* and *IL1B* and increased *IL1RN* expression, which normally inhibits IL-1β activity, would even suggest an anti-inflammatory state. Additionally, *CXCL8, IL1B* and *CLEC4E* were involved in correlation group 4 which included, in contrast to other groups, other downregulated genes. These are known as pro-inflammatory factors (*CCL3, CXCL1, TNF*, etc.).

The least striking changes were observed in macrophages treated with IFNγ. The most interesting was a significant decrease in *ACE* expression depending on HAE severity. *ACE* encodes angiotensin I converting enzyme, a protein responsible for producing angiotensin II as well as bradykinin inactivation (Sheikh and Kaplan, 1986). Inhibiting its activity can induce angioedema outbreak through insufficient bradykinin degradation (Anderson and deShazo, 1990) as observed in hypertension patients medicated with ACE inhibitors (ACEi-AE). However, most HAE patients have a normal ACE level and the influence of locally synthesized enzymes on bradykinin degradation is not known. Individual differences in ACE activity were confirmed for carriers of different ACE alleles (Rigat et al., 1990), but these differences were not associated with increased angioedema risk in ACEi-AE patients (Bas et al., 2010). On the other hand, higher ACE activity accompanies many inflammatory diseases (Cao et al., 2020), although its exact role has not been fully explained. If macrophages stimulated by IFNγ can contribute to the total ACE activity in the site of swelling, decreased ACE expression could be a reason for more serious HAE progress.

## 4 Conclusion

While the modifying effect of identified gene variants on HAE severity has not been proved, transcriptomic analysis of selected myeloid lineage cells showed that some genes were differently expressed and that the overall balance between gene expression depended on HAE severity. In addition, the number of differently expressed genes was elevated in patients with a mild disease course and even more prominently with a severe disease course. It indicated that the steady-state level of some genes in these cells was somehow shifted, similar to the dysregulation observed in HAE patients’ neutrophils (Grymová et al., 2019). Thus, HAE patients’ monocytes/macrophages seem to be susceptible to form, in response to some unknown factor(s), the networks of mutually tightly co-regulated genes which may then have an impact on edema development. Such regulatory intervention is probably not caused by bradykinin-mediated HAE attacks, as bradykinin stimulates *CXCL8* (Koyama et al., 2000) or *MMP9* (Yang et al., 2022) expression which is not apparently the case in HAE patients, and other factors must be considered. IFNγ could be such a factor, a *SERPING1, CXCL10* and *CXCL11* are genes regulated by it. However, our analysis showed that HAE patients had comparable IFNγ levels to the controls, while the inflammatory gene expression seemed to be rather decreased. While monocytes were isolated as soon after taking blood as possible, macrophages were cultivated for 3 days. We assumed that measured mRNAs levels thus reflected the predisposition of *ex vivo* cultivated macrophages rather than actual organism state, while in monocytes both effects could be combined. This can be demonstrated on some genes’ altered expression levels and on the correlation of gene groups 3, 4 in macrophages and 7 in macrophages treated with IFNγ, which is apparently related to HAE severity. Anyway, pro-inflammatory hypothesis about attack predisposition seems not to be correct. Although some results can be understood in a contradictory way, the overall expression pattern in HAE patients was rather anti-inflammatory, which could be related to previous edema attacks and/or reflect patients’ adjustment in a symptom-free period as their preventive measure against edema development.

## 5 Materials and methods

### 5.1 Biological material

A total of 159 HAE patients (OMIM #106100) from the Czech Republic were recruited for DNA analysis, 40 of them plus 20 healthy controls were submitted for transcriptomic analysis. HAE-related data, particularly the age at disease onset, age at diagnosis, number of attacks per year, C1-inhibitor plasma levels, and C1-inhibitor function, were collected both from the Czech national registry of primary immunodeficiencies, where all known diagnosed HAE patients in the Czech Republic are registered, and from attending physicians in 4 Czech HAE centres, who completed individual forms for each patient. The data collected until October 2021 were analysed. The age at disease onset, average number of attacks per year in the last 5 years, maximum number of attacks per year, and, in the case of mRNA analysis, the number of attacks in the last year before blood collection, were considered when sorting patients into their disease severity groups. Data on treatment was derived from 193 HAE patients in the Czech national registry, which reflects the situation in our cohort well. Out of 6,945 attacks recorded in 166 patients between March 2012 and October 2021, 5691 (81.9%) were treated; 3562 of them (62.0%) with icatibant (Firazyr^®^), 847 (14.9%) with rhC1-INH (Ruconest^®^), 1002 (17.6%) with pnfC1-INH (Berinert^®^) and 83 (1.5%) with nfC1-INH (Cynrize^®^); the remaining 134 (2.4%) attacks were treated with attenuated androgens, 88 (1.5%) with tranexamic acid, and 10 (0.2%) with fresh frozen plasma. Fixed drug doses were used as follows: Firazyr^®^ 30 mg, Ruconest^®^ 2,100 U 1–2 vials (median dose 2,100 U, range 2,100–4,200 U), and Berinert^®^ 500 IU 1–2 vials (median dose 1,000 IU, range 500–1,000 IU). Long-term prophylaxis was used in 106 patients. Treatment had to be repeated in 0.2% of attacks. The treatment was evaluated as effective in 98.6% of attacks.

The study was approved by the Medical Ethics Committee of St. Anne’s University Hospital (ethics approval number: 6G/2015, Brno). Informed consent was obtained from all the participants before being included in the study. The study conforms to the Declaration of Helsinki standards. None of the patients/controls displayed symptoms of HAE attack or infectious disease at the time of sampling nor suffered from chronic infectious complications.

### 5.2 Cell isolation

From each patient or control, 15 mL of whole blood was taken in a symptom-free period, and peripheral blood mononuclear cells (PBMCs) were isolated by density gradient centrifugation using Ficoll-Paque (Pharmacia). From the total PBMCs, monocytes were isolated by positive selection with magnetic beads using a Dynabeads FlowComp Human CD14 Kit (Invitrogen). Monocytes were subsequently divided into nine aliquots. Three of them (comprising 200,000 cells each) were frozen directly. The remaining six aliquots (100,000 cells each) were cultivated for 3 days in RPMI medium supplemented with 10% FBS, 100 U/mL penicillin, 100 mg/mL streptomycin, and 2 mM L-glutamine. 100 ng/ml M-CSF (Peprotech) was added to the cells to stimulate their differentiation into macrophages, and three wells were further polarized by adding 5 ng/mL interferon-gamma (IFNγ) for the last 24 h. The incubation was carried out at 37°C and 5% CO2. Each cell type was thus represented by triplicates for each patient/control.

Collected heparinized blood samples were stored at 22°C, and all procedures began at the latest 1 h after blood collection. The viability of isolated monocytes was measured on ViCell XRcell viability analyzer (Beckman Coulter), and the viability ranged from 90% to 95% in all samples. The purity, yield, differentiation and activation of monocytes were determined by flow cytometry measurements. The purity of monocytes was above 95% in all used samples. Monocyte differentiation to monocyte-derived macrophages was determined by CD86 expression, which is typically expressed by antigen presenting cells and increases with the monocyte differentiation. Monocyte-derived macrophages from all samples had a CD86 surface expression over 99% after stimulation with M-CSF or M-CSF + IFNγ for 72 h. Monocyte-derived macrophage activation was determined by CD38 surface expression (marker of cell activation). Monocyte-derived macrophages without stimulation with IFNγ did not express CD38. All monocyte-derived macrophages activated by IFNγ showed a homogenous increase of CD38 expression (gating strategy is summarized in the Supplementary Figure S5).

### 5.3 DNA sequencing

DNA was prepared from 5 mL of freshly drawn sodium-EDTA whole blood using a standard desalting procedure. To prepare DNA libraries, SureSelectXT HS Low Input Reagent Kit (Agilent) was used. In brief, 100 ng of DNA was sheared by an enzymatic kit (Agilent) to preserve DNA fragments at 150 to 200 bp. The next reaction ensured DNA end repair and tailing. Adaptors were ligated overnight and purified by AMPure XP beads (Becman Coulter). The qualities of individual samples were checked on Tape Station (Agilent, D1000 ScreenTape). 12 DNA libraries were pooled to achieve a concentration of 125 ng/μL in 12 µL of solution. The desired DNA fragments (Supplementary Table S2) were acquired using SureSelectXT Custom enrichment kit 0.5–2.9 Mb (Agilent) according to the manufacturer’s instructions. Afterwards, the captured DNA was amplified using a post-capture PCR Reaction mix and purified by AMPure XP beads. The DNA library quality was checked on Tape Station and concentration measured by Qubit dsDNA HS Assay Kit (Thermofisher). In the end, two pooled libraries were pooled together to get a library of 24 individual patients. The acquired DNA libraries were paired-end sequenced by Miseq using MiSeq Reagent Kit v2 (300 cycles) (Illumina).

### 5.4 RNA sequencing

Total RNA was isolated from all cell types by the RNeasy plus mini kit (Qiagen, Hilden, Germany). The RNA quantity was determined by Qubit RNA HS Assay Kit (Thermofisher) and the quality was checked by High Sensitivity RNA ScreenTape on the Tape Station system (Agilent). For the purposes of targeted RNA-seq library preparation, a QIAseq Targeted RNA Custom Panel (Qiagen) was designed and used for transcript quantitation. Appropriate libraries were prepared according to the manufacturer’s instructions. Acquired libraries were pooled and sequenced on an Illumina NextSeq using NextSeq 500/550 Mid Output Kit v2.5 (150 Cycles) as single reads. As the results were acquired from six NextSeq runs, an artificial (mixed) control of RNA isolated from different cell types was used to ensure reproducibility in all these runs.

### 5.5 Quantitative RT-PCR

cDNA was synthesized using random hexamers and RT^2^ First Strand Kit (Qiagen) and the RNA used was the same as in the RNA-seq experiment. Subsequent real time PCR was performed using PrimeTime qPCR Probe Assays (Integrated DNA Technologies). Primer sequences are depicted in Supplementary Table S8, and reaction conditions are available upon request. Three genes used for normalization were selected based on the highest expression stability in the RNA-seq experiment. PCR was performed in technical triplicates for genes of interest and duplicates for normalization genes on a LightCycler 480 Instrument II (Roche). Data quantities were determined using absolute quantitation.

### 5.6 Computational analysis of NGS data

The quality of raw sequence data (for both DNA-seq and RNA-seq) was checked with FastQC combined with MultiQC. DNA reads (paired ends) in fastq format were mapped against the human reference genome hg19 using Burrows-Wheeler Aligner (BWA) (http://bio-bwa.sourceforge.net/). Alignment obtained in sam format was converted to bam, sorted and indexed using SAMtools (http://samtools.sourceforge.net/). Samblaster was used to mark PCR duplicates, which were not used in subsequent analysis. To discover genetic variants, Vardict tool (https://github.com/AstraZeneca-NGS/VarDict) was employed. The obtained variants were restricted by chromosomal regions defined in a custom bed file derived according to the designed gene targets (Supplementary Table S2) and annotated with ANNOVAR (http://annovar.openbioinformatics.org/en/latest/).

RNA reads (single end) were processed using in-house bioinformatics pipeline and available online tools. Trimmomatic was employed to remove adaptor sequences, discard low-quality reads, eliminate poor-quality bases and cut UMI sequences (12 nt at the beginning of reads) into individual fastq files. RNA reads without adaptor and UMI sequences were mapped to the reference human genome GRCh38 using STAR aligner (http://code.google.com/p/rna-star/).

Fgbio toolkit (http://fulcrumgenomics.github.io/fgbio/) was employed to annotate obtained transcriptome bam files with UMI sequences. The annotated bam files were sorted with Samtools and deduplicated with Umitools (https://github.com/CGATOxford/UMI-tools). For gene quantification, featureCounts (http://www.bioconductor.org) was used. Differential expression analysis was conducted using DESeq2 programs implemented in R package (http://www.bioconductor.org).

### 5.7 Statistical analysis

Within DNA-seq analysis, gene variant frequencies were compared using a chi square test. Significant *p*-values were adjusted to value representing Bonferroni correction considering only identified gene variants having overall frequency, which enabled the designated *p*-value to be exceeded in the corresponding chi square test. For other variants, the chi square test was not applied (Supplementary Table S5).

Gene expression differences in RNA-seq datasets were tested with a Wald test implemented in DESeq2 program. The significant *p*-value was adjusted to 3.65E-04 representing a Bonferroni correction of *p*-value 0.05 with respect to the analyzed gene number (136). Every gene showing lower *p*-value was individually inspected to evaluate the outliers’ effect on the output of applied statistical test. In rare cases, the identified outliers were removed from the analyzed groups to minimize the effect of these excesses on the final *p*-value. As a small number of identified reads resulted in biased low *p*-values, an additional discriminating parameter was implemented. Thus, only gene expression comparisons reaching a minimal average read number of 30 for both groups were considered.

The correlation in gene expression was counted as the Pearson correlation coefficient for all gene combinations. The groups of co-expressed genes were defined based on a specific search for the highest correlation coefficients (exceeding 0.7). Final co-expressed gene groups thus meet the following criteria: 1) each gene group contains at least five members. 2) at least one correlation coefficient in each gene reached a value of 0.7 inside this gene group and this criterion could only be met in one CC4 subgroup. Changes in correlation coefficients were evaluated by Wilcoxon test.

The difference in gene expression acquired by quantitative RT-PCR was tested using the Mann-Whitney test.

## Data Availability

The datasets presented in this article are not readily available because of concerns regarding patient privacy and consent. Requests to access the datasets should be directed to the corresponding authors.
