# Supplementary material for: Myeloid lineage cells evince distinct steady-state level of certain gene groups in dependence on hereditary angioedema severity
Source: Front Genet. 2023 Jul 4;14:1123914. doi: 10.3389/fgene.2023.1123914 (PMC10352584; doi:10.3389/fgene.2023.1123914)
Supplement: Supplementary file 1 [file DataSheet1.PDF]

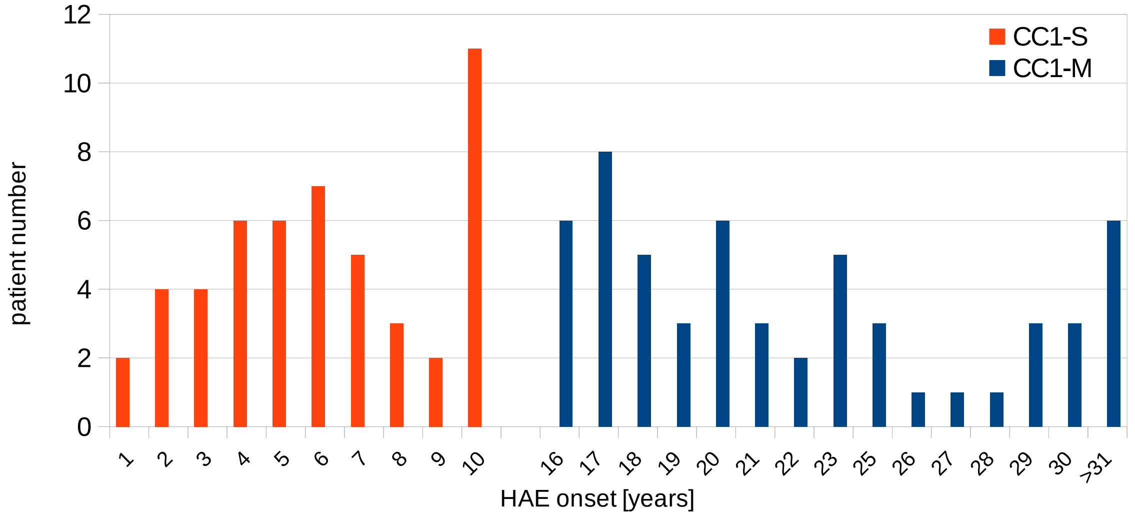

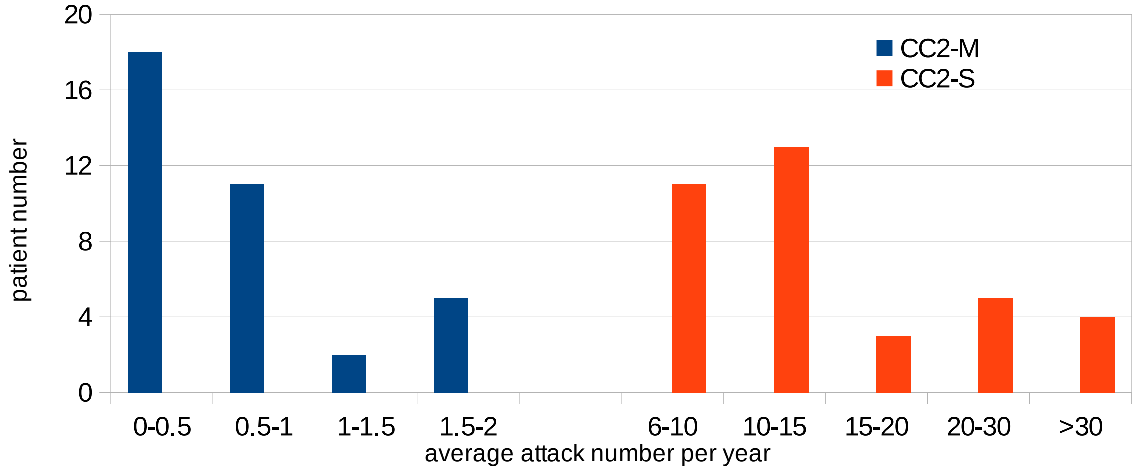

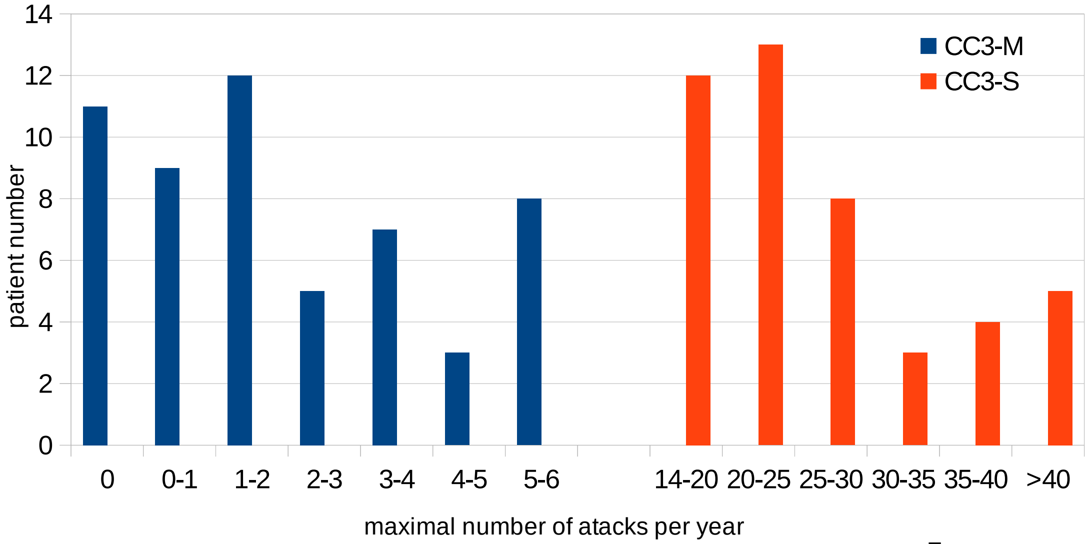

Supplementary figure S1. Patient distribution in CC groups according to the appropriate criterion.

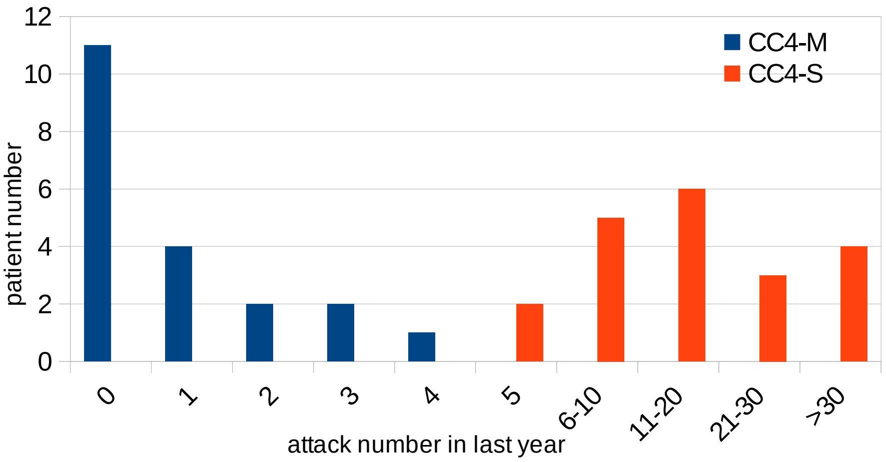

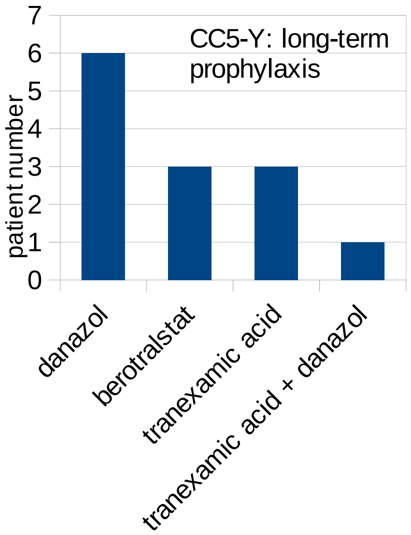

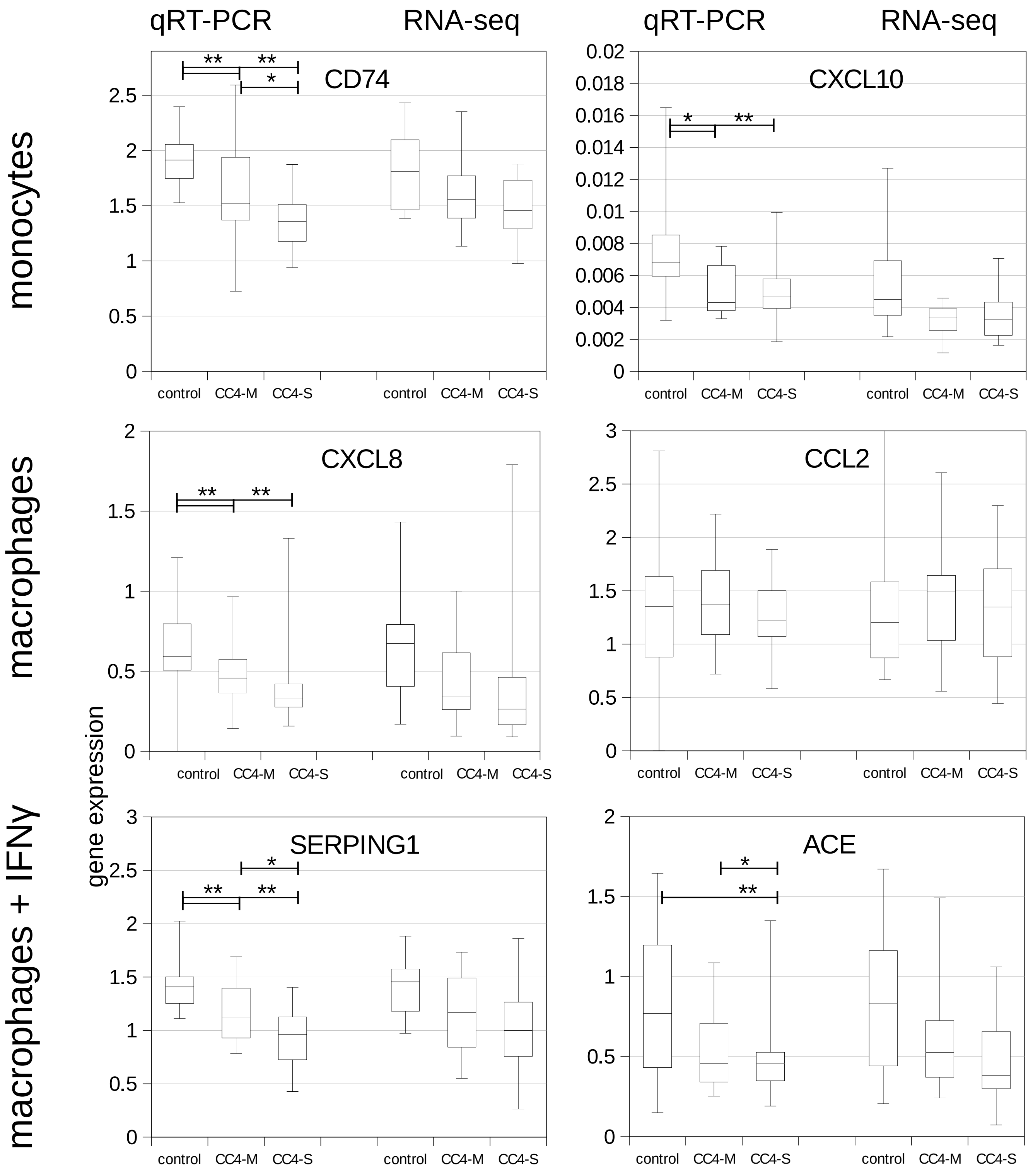

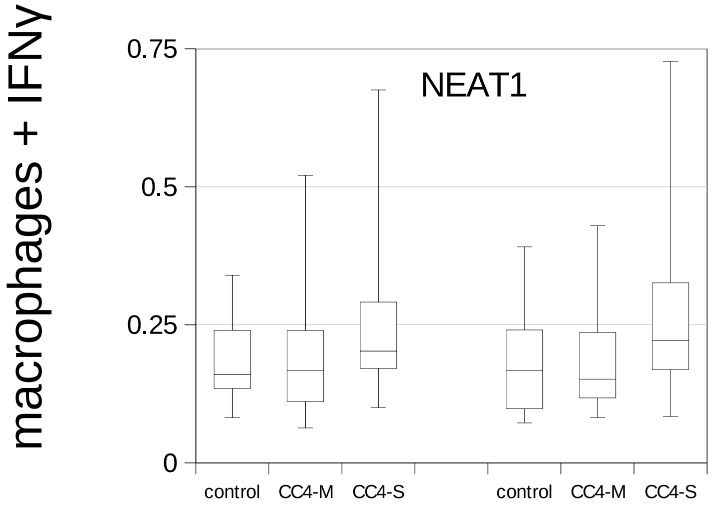

Supplementary figure S2. Comparison of gene expression by RNA-seq and qRT-PCR. Gene expression is depicted in artificial units and presented as boxplots. Differences in gene expression (qRT-PCR) were tested by Mann-Whitney test. * $p < 0.05$. ** $p < 0.01$.

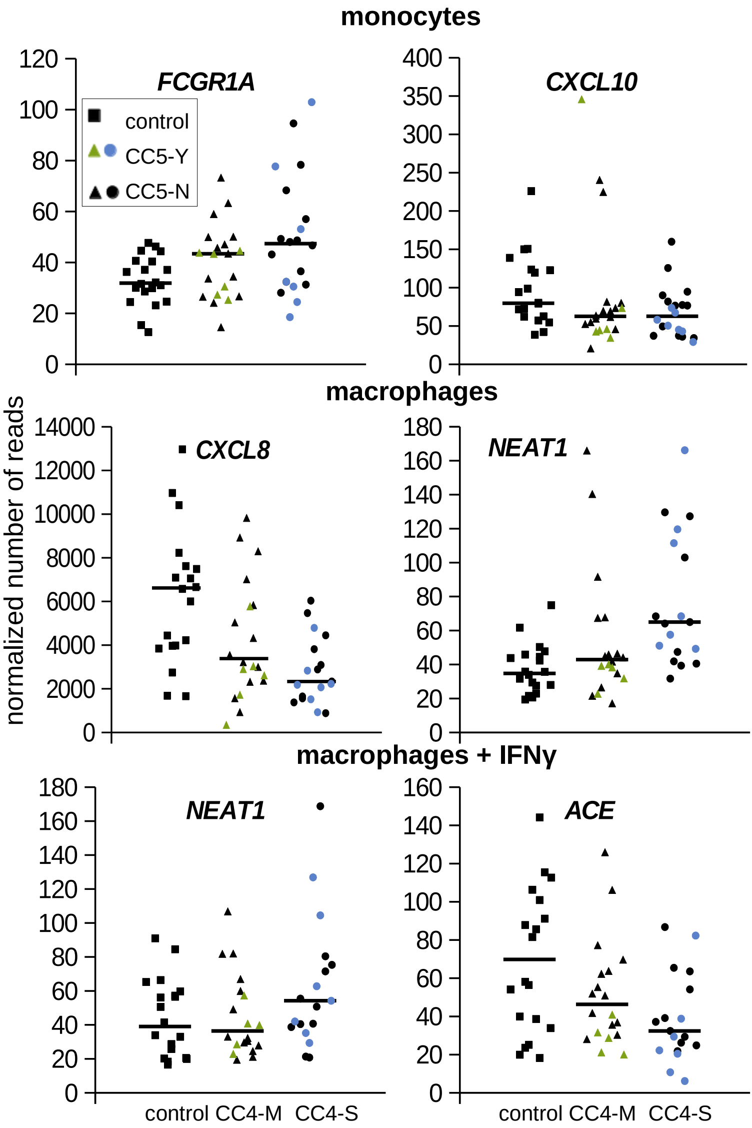

Supplementary figure S3. The infuence of long-term profylaxis on transcript levels. Plots depict normalized read counts for controls and HAE patients categorized to CC4-M/ CC4-S (columns) and CC5-Y(coloured points)/CC5-N (black points). Horizontal bars represent median.

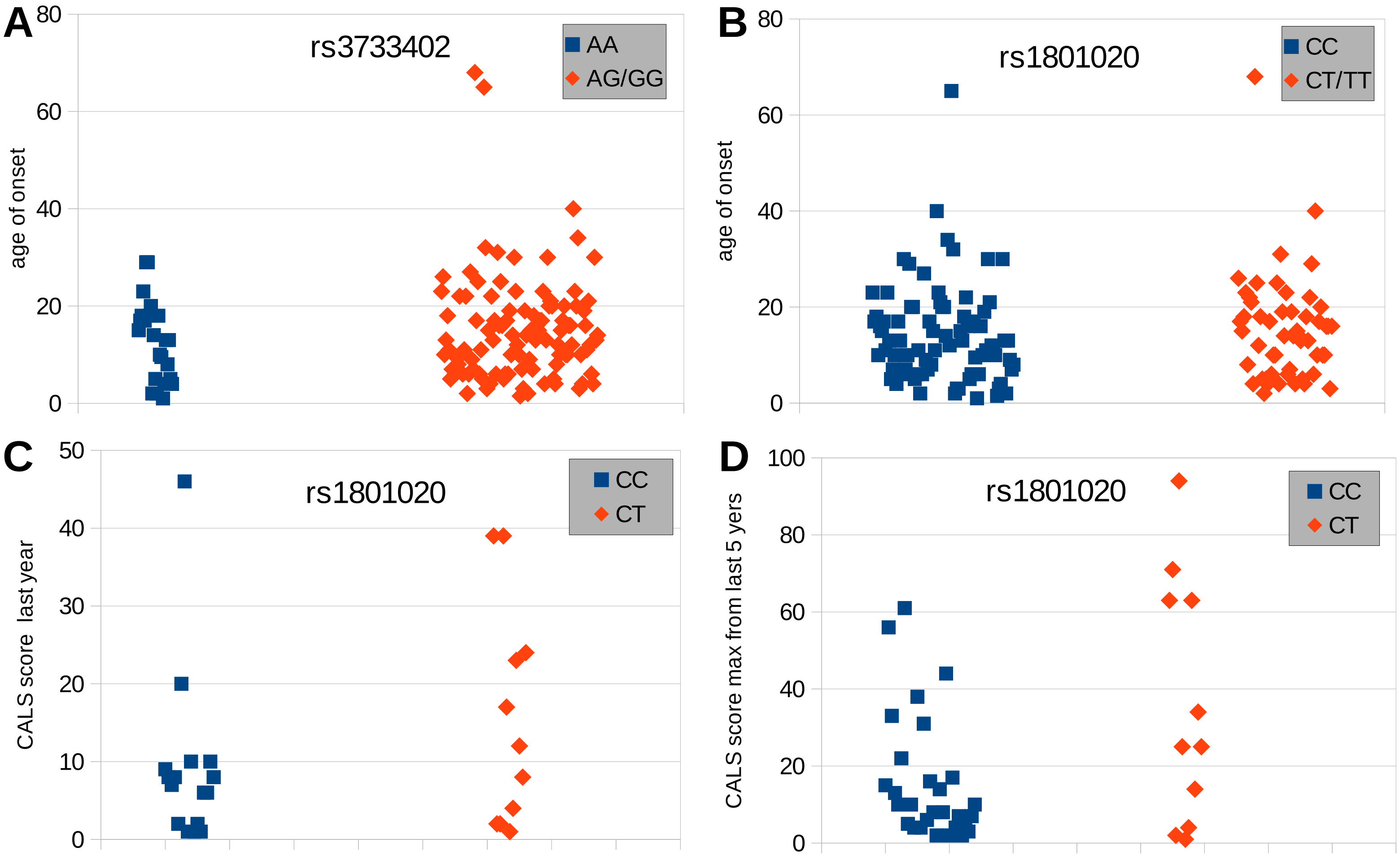

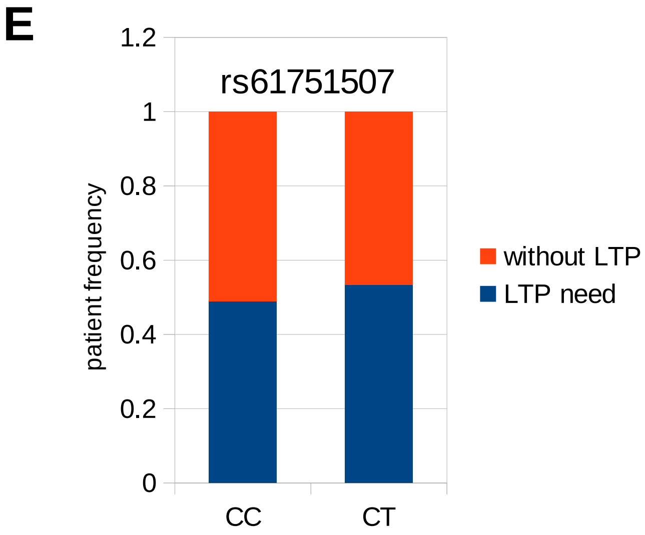

Supplementary figure S4. Association of selected variants in *KLKB1* (A), *F12* (B-D) and *CPN1* (E) genes with HAE phenotype. Particular variants and phenotypic characteristics were chosen based on studies by Bors et al., 2013; Gianni et al., 2017; and Parsopoulou et al., 2022). (A-B) Average age of HAE onset, (C-D) CALS score and (E) a frequency of patient with a need of long-term prophylaxis was not significantly different for carriers of particular gene variants. 1-year CALS score was counted according to Parsopoulou et al., 2022 (C) in last year preceding data collection, or (D) in last 5 years with subsequent selection of the highest value.

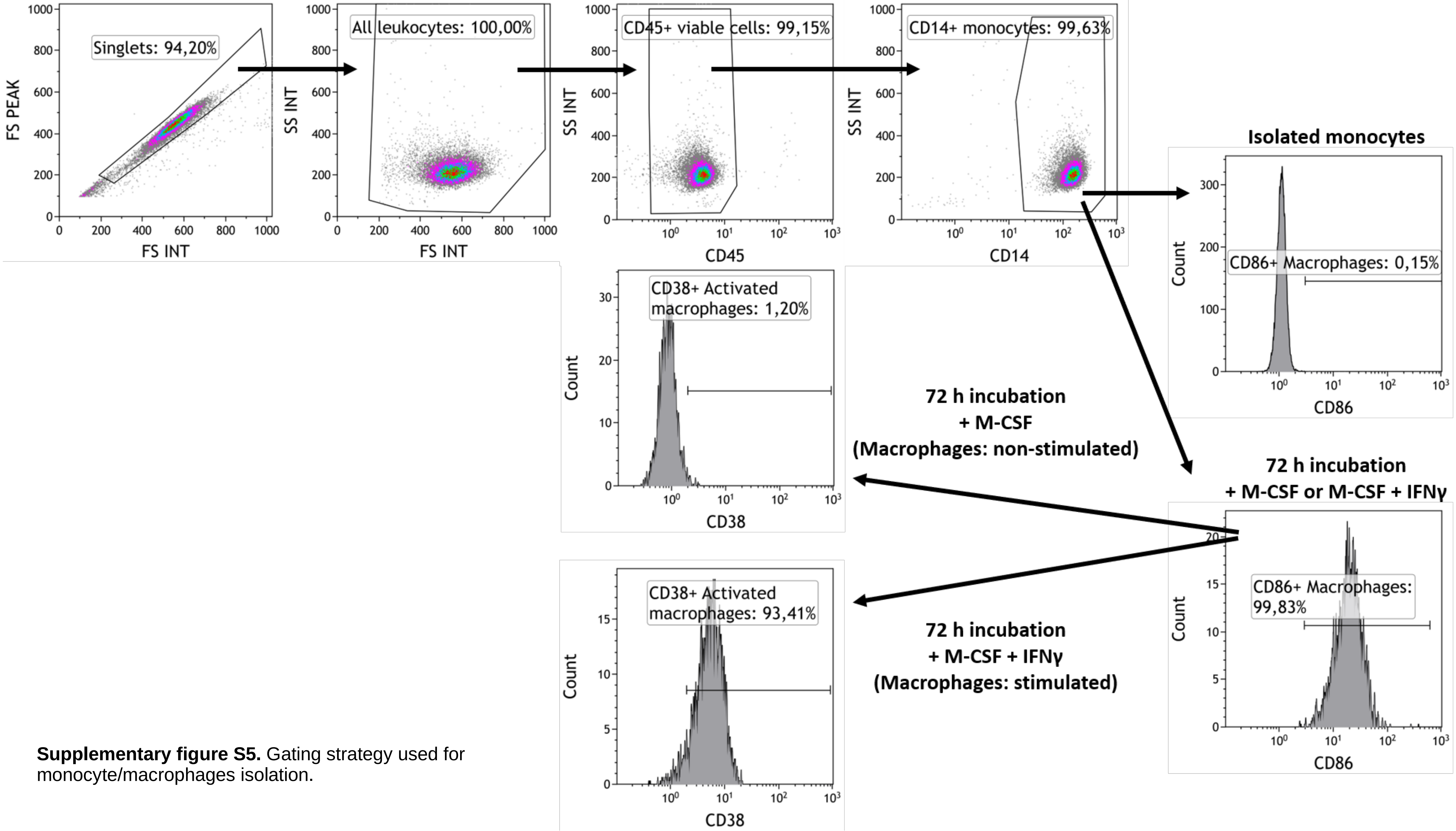

Supplementary figure S5. Gating strategy used for monocyte/macrophages isolation.

monocytes

control		corr_group_1A																	corr_group_1B														
		TLR4	PRCP	IFNAR1	IFNGR1	CD36	C1QBP	HSP90AB1	HSP90AA1	IMMT	IFNGR2	CASP1	IL1RN	MYD88	CPM	TRAF6	TNF	CXCR2	TRAF1	REL	IKBKB	NEAT1	NLRP3	RBM15	IRAK3	SEMA4D	XPNPEP1	KIAA0586	NFKB2	GBP2	TNFAIP2	ARRB2	ZBTB22
corr_group_1A	TLR4	-																															
	PRCP	0.9	-																														
	IFNAR1	0.8	0.8	-																													
	IFNGR1	0.6	0.6	0.4	-																												
	CD36	0.3	0.4	0.3	0.5	-																											
	C1QBP	0.6	0.7	0.6	0.5	0.7	-																										
	HSP90AB1	0.8	0.8	0.8	0.4	0.2	0.5	-																									
	HSP90AA1	0.6	0.6	0.7	0.4	0.4	0.6	0.6	-																								
	IMMT	0.7	0.7	0.8	0.3	0.4	0.8	0.7	0.7	-																							
	IFNGR2	0.6	0.8	0.8	0.4	0.5	0.7	0.8	0.6	0.8	-																						
	CASP1	0.4	0.5	0.5	0.6	0.5	0.7	0.4	0.7	0.6	0.6	-																					
	IL1RN	0.3	0.5	0.4	0.3	0.6	0.6	0.3	0.3	0.5	0.6	0.4	-																				
	MYD88	0.4	0.6	0.5	0.6	0.8	0.8	0.4	0.7	0.7	0.6	0.7	0.5	-																			
	CPM	0.7	0.7	0.7	0.6	0.4	0.7	0.7	0.6	0.7	0.7	0.5	0.4	0.6	-																		
	TRAF6	0.3	0.4	0.4	0.4	0.6	0.5	0.4	0.6	0.4	0.5	0.4	0.4	0.6	0.5	-																	
	TNF	0.6	0.7	0.8	0.4	0.4	0.7	0.6	0.5	0.7	0.7	0.6	0.5	0.6	0.4	0.4	-																
	CXCR2	0.6	0.7	0.5	0.3	0.5	0.7	0.6	0.4	0.6	0.6	0.4	0.6	0.5	0.6	0.4	0.3	-															
corr_group_1B	TRAF1	-0.5	-0.6	-0.3	-0.4	-0.3	-0.5	-0.3	-0.4	-0.4	-0.2	-0.5	-0.3	-0.5	-0.3	-0.2	-0.2	-0.5	-														
	REL	-0.6	-0.7	-0.5	-0.3	0.0	-0.4	-0.6	-0.3	-0.5	-0.4	-0.3	-0.2	-0.4	-0.4	0.0	-0.3	-0.5	0.6	-													
	IKBKB	-0.4	-0.5	-0.4	-0.6	-0.7	-0.8	-0.3	-0.6	-0.5	-0.5	-0.7	-0.6	-0.8	-0.5	-0.4	-0.5	-0.5	0.6	0.3	-												
	NEAT1	-0.7	-0.7	-0.6	-0.4	-0.3	-0.6	-0.6	-0.3	-0.6	-0.4	-0.3	-0.3	-0.4	-0.5	-0.1	-0.3	-0.6	0.7	0.8	0.4	-											
	NLRP3	-0.5	-0.5	-0.4	-0.5	-0.5	-0.7	-0.3	-0.4	-0.5	-0.4	-0.6	-0.4	-0.7	-0.6	-0.3	-0.3	-0.5	0.6	0.5	0.7	0.7	-										
	RBM15	-0.7	-0.7	-0.7	-0.5	-0.2	-0.5	-0.6	-0.5	-0.6	-0.5	-0.4	-0.4	-0.4	-0.6	-0.3	-0.6	-0.4	0.4	0.7	0.5	0.6	0.5	-									
	IRAK3	-0.5	-0.6	-0.3	-0.4	-0.3	-0.4	-0.4	-0.2	-0.4	-0.4	-0.3	-0.4	-0.3	-0.2	-0.1	-0.5	-0.4	0.5	0.6	0.4	0.6	0.3	0.6	-								
	SEMA4D	-0.4	-0.3	-0.3	-0.5	-0.4	-0.5	-0.3	-0.3	-0.4	-0.3	-0.2	-0.3	-0.5	-0.5	-0.5	-0.3	-0.4	0.2	0.3	0.3	0.6	0.6	0.4	0.3	-							
	XPNPEP1	-0.5	-0.4	-0.2	-0.2	0.0	-0.2	-0.2	-0.1	-0.2	0.0	0.0	0.0	0.0	-0.2	0.1	-0.1	-0.3	0.5	0.6	0.1	0.7	0.2	0.5	0.6	0.2	-						
	KIAA0586	-0.3	-0.4	-0.2	-0.5	0.0	-0.3	-0.3	-0.1	-0.2	-0.2	-0.4	-0.2	-0.2	-0.1	0.0	-0.4	-0.2	0.5	0.4	0.4	0.3	0.1	0.3	0.5	0.0	0.3	-					
	NFKB2	-0.4	-0.4	-0.3	-0.5	-0.5	-0.6	-0.2	-0.3	-0.5	-0.4	-0.5	-0.5	-0.7	-0.6	-0.4	-0.3	-0.6	0.5	0.4	0.6	0.5	0.8	0.2	0.2	0.6	0.0	0.0	-				
	GBP2	-0.3	-0.3	-0.2	0.1	-0.2	-0.3	-0.3	-0.1	-0.4	-0.2	0.2	-0.2	-0.2	-0.4	-0.2	0.0	-0.5	0.1	0.4	0.0	0.5	0.2	0.2	0.1	0.4	0.4	-0.2	0.2	-			
	TNFAIP2	-0.4	-0.5	-0.3	-0.6	-0.6	-0.7	-0.2	-0.4	-0.4	-0.4	-0.6	-0.5	-0.8	-0.6	-0.4	-0.3	-0.6	0.7	0.4	0.9	0.5	0.9	0.3	0.3	0.5	0.2	0.2	0.8	0.2	-		
	ARRB2	-0.5	-0.4	-0.2	-0.3	0.0	-0.1	-0.2	-0.1	0.0	0.0	0.0	-0.1	0.0	-0.1	0.1	0.0	-0.2	0.5	0.5	0.2	0.5	0.4	0.3	0.3	0.2	0.5	0.2	0.3	-0.1	0.4	-	
	ZBTB22	-0.7	-0.7	-0.8	-0.5	-0.3	-0.6	-0.7	-0.5	-0.6	-0.8	-0.6	-0.4	-0.5	-0.6	-0.3	-0.7	-0.5	0.4	0.5	0.5	0.5	0.4	0.5	0.3	0.2	0.0	0.4	0.5	0.0	0.4	0.3	-

CC4-M		corr_group_1A																	corr_group_1B														
corr_group_1A	TLR4	-																															
	PRCP	0.9	-																														
	IFNAR1	0.9	0.9	-																													
	IFNGR1	0.8	0.9	0.8	-																												
	CD36	0.8	0.7	0.8	0.8	-																											
	C1QBP	0.7	0.7	0.7	0.5	0.6	-																										
	HSP90AB1	0.7	0.6	0.7	0.4	0.4	0.7	-																									
	HSP90AA1	0.8	0.6	0.7	0.5	0.5	0.8	0.7	-																								
	IMMT	0.6	0.6	0.7	0.4	0.5	0.9	0.7	0.8	-																							
	IFNGR2	0.7	0.6	0.6	0.4	0.7	0.7	0.5	0.6	0.7	-																						
	CASP1	0.6	0.6	0.6	0.4	0.4	0.9	0.6	0.8	0.8	0.6	-																					
	IL1RN	0.6	0.7	0.7	0.7	0.7	0.4	0.3	0.4	0.4	0.5	0.4	-																				
	MYD88	0.4	0.4	0.4	0.4	0.5	0.7	0.3	0.6	0.6	0.5	0.6	0.4	-																			
	CPM	0.4	0.4	0.4	0.4	0.2	0.5	0.5	0.5	0.5	0.2	0.3	0.1	0.4	-																		
	TRAF6	0.6	0.6	0.6	0.7	0.8	0.5	0.3	0.5	0.5	0.5	0.2	0.6	0.4	0.3	-																	
	TNF	0.5	0.5	0.5	0.4	0.5	0.6	0.4	0.5	0.6	0.7	0.5	0.5	0.6	0.2	0.4	-																
	CXCR2	0.2	0.3	0.4	0.2	0.3	0.6	0.3	0.4	0.7	0.7	0.6	0.3	0.6	0.3	0.3	0.7	-															
corr_group_1B	TRAF1	-0.4	-0.4	-0.4	-0.3	-0.4	-0.6	-0.3	-0.4	-0.5	-0.4	-0.5	-0.4	-0.7	-0.1	-0.4	-0.4	-0.4	-														
	REL	-0.4	-0.4	-0.4	-0.2	-0.3	-0.5	-0.5	-0.4	-0.5	-0.4	-0.6	-0.3	-0.6	-0.1	-0.1	-0.5	-0.5	0.8	-													
	IKBKB	-0.3	-0.3	-0.3	-0.3	-0.4	-0.6	-0.3	-0.4	-0.4	-0.3	-0.4	-0.3	-0.8	-0.2	-0.4	-0.5	-0.5	0.8	0.6	-												
	NEAT1	-0.5	-0.5	-0.5	-0.3	-0.4	-0.7	-0.5	-0.5	-0.6	-0.4	-0.7	-0.3	-0.8	-0.4	-0.2	-0.6	-0.7	0.7	0.8	0.7	-											
	NLRP3	-0.3	-0.2	-0.3	-0.2	-0.3	-0.5	-0.3	-0.4	-0.4	-0.3	-0.4	-0.2	-0.7	-0.3	-0.3	-0.4	-0.5	0.7	0.7	0.8	0.8	-										
	RBM15	-0.4	-0.5	-0.5	-0.3	-0.4	-0.5	-0.4	-0.3	-0.4	-0.4	-0.5	-0.4	-0.6	0.0	-0.2	-0.6	-0.5	0.8	0.9	0.7	0.7	0.7	-									
	IRAK3	-0.3	-0.4	-0.4	-0.1	-0.4	-0.5	-0.4	-0.4	-0.5	-0.5	-0.5	-0.4	-0.6	0.1	-0.3	-0.6	-0.6	0.8	0.9	0.7	0.7	0.7	0.8	-								
	SEMA4D	-0.2	-0.2	-0.3	-0.2	-0.3	-0.5	-0.3	-0.3	-0.4	-0.2	-0.4	-0.2	-0.7	-0.1	-0.2	-0.5	-0.5	0.9	0.7	0.9	0.7	0.8	0.8	0.8	-							
	XPNPEP1	-0.5	-0.5	-0.4	-0.4	-0.4	-0.4	-0.3	-0.3	-0.4	-0.3	-0.5	-0.4	-0.5	0.0	-0.3	-0.4	-0.4	0.8	0.8	0.7	0.7	0.7	0.9	0.8	0.7	-						
	KIAA0586	-0.3	-0.3	-0.3	-0.2	-0.2	-0.5	-0.3	-0.2	-0.3	-0.2	-0.5	-0.3	-0.5	0.1	-0.1	-0.4	-0.4	0.8	0.8	0.7	0.5	0.5	0.8	0.8	0.8	0.8	-					
	NFKB2	-0.1	-0.2	-0.2	-0.2	-0.3	-0.6	-0.1	-0.3	-0.5	-0.2	-0.4	-0.3	-0.8	-0.2	-0.3	-0.4	-0.6	0.8	0.6	0.8	0.7	0.8	0.6	0.7	0.8	0.5	0.6	-				
	GBP2	-0.3	-0.3	-0.3	-0.3	-0.5	-0.4	-0.1	-0.2	-0.3	-0.3	-0.3	-0.3	-0.6	-0.1	-0.4	-0.4	-0.5	0.7	0.6	0.7	0.6	0.8	0.6	0.7	0.8	0.6	0.5	0.6	-			
	TNFAIP2	-0.5	-0.5	-0.5	-0.5	-0.5	-0.6	-0.3	-0.5	-0.4	-0.3	-0.4	-0.4	-0.8	-0.4	-0.5	-0.4	-0.5	0.7	0.4	0.8	0.7	0.7	0.5	0.5	0.8	0.5	0.4	0.8	0.7	-		
	ARRB2	-0.6	-0.5	-0.6	-0.5	-0.6	-0.5	-0.5	-0.5	-0.4	-0.4	-0.4	-0.5	-0.6	-0.2	-0.5	-0.5	-0.3	0.7	0.6	0.7	0.6	0.7	0.7	0.6	0.7	0.7	0.5	0.5	0.7	0.7	-	
	ZBTB22	-0.7	-0.6	-0.8	-0.5	-0.7	-0.7	-0.6	-0.7	-0.7	-0.7	-0.7	-0.7	-0.6	-0.3	-0.5	-0.6	-0.6	0.6	0.7	0.4	0.7	0.5	0.6	0.7	0.5	0.6	0.5	0.5	0.5	0.5	0.6	-

CC4-S		corr_group_1A																	corr_group_1B														
corr_group_1A	TLR4	-																															
	PRCP	0.8	-																														
	IFNAR1	0.6	0.8	-																													
	IFNGR1	0.6	0.7	0.6	-																												
	CD36	0.7	0.6	0.4	0.8	-																											
	C1QBP	0.6	0.6	0.7	0.5	0.4	-																										
	HSP90AB1	0.6	0.5	0.4	0.2	0.2	0.5	-																									
	HSP90AA1	0.5	0.1	0.2	0.1	0.1	0.5	0.4	-																								
	IMMT	0.3	0.2	0.4	0.3	0.2	0.7	0.4	0.5	-																							
	IFNGR2	0.5	0.3	0.5	0.5	0.4	0.6	0.4	0.7	0.6	-																						
	CASP1	0.5	0.4	0.5	0.4	0.3	0.7	0.4	0.6	0.7	0.6	-																					
	IL1RN	0.3	0.3	0.5	0.5	0.6	0.3	0.0	0.0	0.2	0.4	0.1	-																				
	MYD88	0.5	0.5	0.6	0.7	0.5	0.7	0.4	0.5	0.6	0.8	0.6	0.4	-																			
	CPM	0.6	0.7	0.8	0.6	0.4	0.8	0.5	0.2	0.6	0.5	0.6	0.4	0.7	-																		
	TRAF6	0.5	0.2	0.4	0.3	0.2	0.5	0.4	0.6	0.5	0.6	0.4	0.1	0.5	0.4	-																	
	TNF	0.4	0.4	0.4	0.2	0.3	0.6	0.4	0.4	0.4	0.5	0.5	0.1	0.4	0.4	0.3	-																
	CXCR2	0.5	0.6	0.7	0.8	0.5	0.6	0.3	0.3	0.5	0.6	0.5	0.5	0.8	0.7	0.5	0.3	-															
corr_group_1B	TRAF1	-0.6	-0.6	-0.6	-0.5	-0.5	-0.5	-0.2	-0.4	-0.2	-0.4	-0.4	-0.4	-0.6	-0.5	-0.4	-0.4	-0.6	-														
	REL	-0.4	-0.5	-0.4	-0.3	-0.4	-0.3	-0.3	-0.2	-0.2	-0.3	-0.2	-0.3	-0.5	-0.4	-0.1	-0.6	-0.4	0.5	-													
	IKBKB	-0.6	-0.7	-0.7	-0.6	-0.5	-0.8	-0.3	-0.4	-0.5	-0.5	-0.6	-0.4	-0.8	-0.7	-0.3	-0.6	-0.7	0.8	0.6	-												
	NEAT1	-0.4	-0.6	-0.5	-0.5	-0.3	-0.5	-0.4	-0.2	-0.3	-0.4	-0.5	-0.3	-0.6	-0.6	-0.3	-0.3	-0.6	0.6	0.5	0.7	-											
	NLRP3	-0.4	-0.6	-0.7	-0.6	-0.4	-0.6	-0.2	-0.4	-0.4	-0.5	-0.6	-0.5	-0.7	-0.7	-0.3	-0.4	-0.7	0.7	0.6	0.8	0.6	-										
	RBM15	-0.3	-0.5	-0.4	-0.5	-0.4	-0.4	-0.2	0.0	-0.3	-0.3	-0.3	-0.2	-0.6	-0.5	0.0	-0.5	-0.6	0.4	0.6	0.7	0.7	0.5	-									
	IRAK3	-0.1	-0.3	-0.4	-0.3	-0.2	-0.3	-0.2	-0.3	-0.1	-0.3	-0.3	-0.3	-0.4	-0.2	-0.2	-0.5	-0.4	0.5	0.6	0.5	0.6	0.6	0.5	-								
	SEMA4D	-0.5	-0.7	-0.7	-0.7	-0.4	-0.6	-0.2	-0.2	-0.3	-0.4	-0.4	-0.5	-0.7	-0.7	-0.3	-0.4	-0.7	0.8	0.6	0.9	0.7	0.9	0.6	0.6	-							
	XPNPEP1	-0.5	-0.5	-0.6	-0.5	-0.4	-0.5	-0.2	-0.3	-0.3	-0.3	-0.3	-0.4	-0.6	-0.6	-0.3	-0.2	-0.6	0.7	0.6	0.7	0.6	0.8	0.4	0.4	0.8	-						
	KIAA0586	-0.3	-0.5	-0.2	-0.2	-0.3	-0.2	-0.3	0.1	0.0	0.0	-0.1	0.0	-0.2	-0.2	0.2	-0.4	-0.2	0.3	0.6	0.4	0.5	0.2	0.7	0.5	0.4	0.3	-					
	NFKB2	-0.4	-0.7	-0.6	-0.6	-0.3	-0.5	-0.2	-0.2	-0.3	-0.3	-0.6	-0.2	-0.7	-0.7	-0.2	-0.3	-0.7	0.7	0.5	0.8	0.8	0.8	0.7	0.5	0.8	0.6	0.4	-				
	GBP2	-0.2	-0.4	-0.4	-0.4	-0.5	-0.2	-0.1	0.3	-0.1	-0.1	0.1	-0.7	-0.3	-0.4	0.0	0.0	-0.3	0.4	0.3	0.4	0.4	0.4	0.2	0.1	0.5	0.4	0.2	0.3	-			
	TNFAIP2	-0.7	-0.7	-0.8	-0.6	-0.5	-0.7	-0.3	-0.5	-0.4	-0.5	-0.6	-0.5	-0.7	-0.7	-0.5	-0.4	-0.7	0.9	0.5	0.9	0.6	0.9	0.4	0.4	0.9	0.8	0.2	0.8	0.4	-		
	ARRB2	-0.6	-0.5	-0.7	-0.5	-0.5	-0.6	-0.4	-0.5	-0.4	-0.6	-0.5	-0.5	-0.7	-0.6	-0.5	-0.5	-0.6	0.7	0.6	0.8	0.5	0.8	0.3	0.4	0.7	0.7	0.1	0.5	0.4	0.9	-	
	ZBTB22	-0.5	-0.2	-0.1	-0.3	-0.4	-0.3	-0.3	-0.5	-0.2	-0.5	-0.4	-0.2	-0.5	-0.2	-0.4	-0.4	-0.3	0.3	0.4	0.4	0.4	0.3	0.3	0.3	0.2	0.4	0.3	0.3	-0.1	0.4	0.5	-

monocytes

control		IL1B	CXCL8	CXCL2	TNFAIP3	CCL3	CCL3L3	IL32	IL7R	PTGS2
		corr_group_2								
corr_group_2	IL1B	-								
	CXCL8	0.4	-							
	CXCL2	0.2	0.7	-						
	TNFAIP3	-0.3	0.2	0.6	-					
	CCL3	0.6	0.5	0.2	-0.3	-				
	CCL3L3	0.5	0.5	0.2	-0.4	0.7	-			
	IL32	0.0	0.2	0.0	-0.1	0.2	0.0	-		
	IL7R	0.2	0.3	0.3	0.2	0.1	0.0	0.0	-	
	PTGS2	0.8	0.4	0.2	-0.2	0.4	0.4	0.0	0.2	-

CC4-M		corr_group_2								
corr_group_2	IL1B	-								
	CXCL8	0.3	-							
	CXCL2	0.1	0.6	-						
	TNFAIP3	-0.2	0.1	0.5	-					
	CCL3	0.7	0.4	0.1	-0.4	-				
	CCL3L3	0.4	0.3	0.3	-0.3	0.8	-			
	IL32	0.0	-0.4	-0.3	0.0	0.0	-0.2	-		
	IL7R	0.0	-0.4	-0.2	0.1	-0.1	-0.3	0.7	-	
	PTGS2	0.5	0.4	0.1	-0.2	0.4	0.1	-0.1	-0.2	-

CC4-S		corr_group_2								
corr_group_2	IL1B	-								
	CXCL8	0.8	-							
	CXCL2	0.9	0.9	-						
	TNFAIP3	0.7	0.7	0.8	-					
	CCL3	0.8	0.8	0.8	0.7	-				
	CCL3L3	0.7	0.8	0.7	0.5	0.8	-			
	IL32	0.6	0.6	0.8	0.7	0.8	0.4	-		
	IL7R	0.5	0.7	0.6	0.7	0.5	0.3	0.7	-	
	PTGS2	0.7	0.6	0.6	0.4	0.3	0.5	0.3	0.3	-

Table S7_correlations

macrophages

control		corr_group_3A														corr_group_3B															
		CD180	GPBAR1	TRAF6	IL10	CD14	CD36	TNFSF13B	TCEAL1	DCAF10	TLR4	CPM	CASP4	IRAK4	CASP1	SERPINE1	NFKB2	TRAF2	TRAF1	ACE	TNFAIP2	IL7R	TNFSF14	HSP90AB1	ITGAM	CSF1	CCL24	CD274	IL17RA	MMP9	IKBKB
corr_group_3A	CD180	-																													
	GPBAR1	0.5	-																												
	TRAF6	0.3	0.5	-																											
	IL10	0.6	0.6	0.4	-																										
	CD14	0.6	0.6	0.2	0.7	-																									
	CD36	0.3	0.5	0.5	0.4	0.4	-																								
	TNFSF13B	0.6	0.5	0.5	0.6	0.5	0.6	-																							
	TCEAL1	0.5	0.5	0.4	0.5	0.6	0.6	0.6	-																						
	DCAF10	0.1	0.2	0.4	0.5	0.1	0.0	0.0	0.2	-																					
	TLR4	0.3	0.5	0.5	0.4	0.2	0.6	0.5	0.2	0.1	-																				
	CPM	0.2	-0.1	0.1	0.2	-0.1	0.0	0.2	0.2	0.2	-0.1	-																			
	CASP4	0.5	0.4	0.3	0.7	0.6	0.2	0.4	0.6	0.4	-0.1	0.3	-																		
	IRAK4	0.2	0.4	0.4	0.4	0.2	0.0	-0.1	0.2	0.6	0.0	0.1	0.5	-																	
	CASP1	0.6	0.4	0.5	0.7	0.5	0.3	0.6	0.6	0.4	0.3	0.3	0.7	0.5	-																
corr_group_3B	SERPINE1	-0.6	-0.6	-0.4	-0.7	-0.7	-0.5	-0.7	-0.6	0.0	-0.6	0.1	-0.5	-0.2	-0.6	-															
	NFKB2	-0.6	-0.6	-0.5	-0.7	-0.6	-0.5	-0.5	-0.6	-0.4	-0.3	-0.3	-0.7	-0.5	-0.6	0.5	-														
	TRAF2	-0.6	-0.6	-0.5	-0.7	-0.7	-0.6	-0.5	-0.6	-0.3	-0.4	-0.1	-0.7	-0.5	-0.7	0.7	0.9	-													
	TRAF1	-0.6	-0.6	-0.5	-0.8	-0.8	-0.5	-0.5	-0.6	-0.4	-0.5	0.0	-0.6	-0.4	-0.6	0.7	0.8	0.8	-												
	ACE	-0.6	-0.6	-0.6	-0.6	-0.6	-0.6	-0.7	-0.6	-0.1	-0.5	-0.2	-0.4	-0.2	-0.6	0.7	0.7	0.7	0.7	-											
	TNFAIP2	-0.5	-0.4	-0.5	-0.5	-0.5	-0.4	-0.3	-0.6	-0.4	-0.1	-0.4	-0.7	-0.6	-0.6	0.4	0.9	0.8	0.6	0.6	-										
	IL7R	-0.6	-0.5	-0.4	-0.4	-0.4	-0.7	-0.7	-0.5	0.2	-0.7	0.0	-0.1	0.2	-0.3	0.7	0.4	0.5	0.6	0.7	0.2	-									
	TNFSF14	-0.6	-0.7	-0.4	-0.7	-0.8	-0.5	-0.5	-0.5	-0.2	-0.6	0.2	-0.4	-0.2	-0.5	0.8	0.5	0.7	0.8	0.6	0.4	0.6	-								
	HSP90AB1	-0.5	-0.4	-0.1	-0.4	-0.5	-0.4	-0.5	-0.3	0.3	-0.5	0.2	-0.1	0.2	-0.3	0.7	0.2	0.4	0.5	0.7	0.1	0.7	0.7	-							
	ITGAM	-0.3	-0.4	-0.3	-0.5	-0.7	-0.5	-0.3	-0.5	-0.2	-0.3	0.2	-0.5	-0.4	-0.5	0.6	0.4	0.6	0.6	0.6	0.5	0.3	0.6	0.5	-						
	CSF1	-0.5	-0.5	-0.3	-0.7	-0.6	-0.3	-0.4	-0.3	-0.2	-0.5	0.2	-0.4	-0.3	-0.5	0.7	0.3	0.5	0.6	0.5	0.2	0.4	0.8	0.6	0.6	-					
	CCL24	-0.6	-0.5	-0.4	-0.6	-0.6	-0.4	-0.4	-0.4	-0.2	-0.5	0.0	-0.4	-0.3	-0.5	0.7	0.6	0.7	0.7	0.7	0.5	0.5	0.8	0.6	0.6	0.8	-				
	CD274	-0.5	-0.5	-0.3	-0.5	-0.6	-0.5	-0.5	-0.3	0.1	-0.7	0.4	-0.1	0.1	-0.2	0.7	0.2	0.5	0.6	0.6	0.1	0.7	0.8	0.8	0.6	0.8	0.7	-			
	IL17RA	-0.6	-0.4	-0.2	-0.6	-0.7	-0.3	-0.4	-0.5	0.0	-0.3	0.1	-0.4	-0.1	-0.5	0.7	0.5	0.6	0.7	0.6	0.4	0.6	0.8	0.6	0.6	0.7	0.8	0.7	-		
	MMP9	-0.4	-0.2	-0.4	-0.1	-0.1	-0.1	-0.3	-0.2	-0.2	-0.1	-0.3	-0.2	-0.2	-0.3	0.1	0.5	0.3	0.3	0.4	0.4	0.3	0.1	0.1	0.0	0.0	0.1	0.0	0.1	-	
	IKBKB	-0.1	-0.2	-0.4	-0.3	-0.1	-0.3	-0.1	-0.4	-0.5	0.1	-0.5	-0.6	-0.5	-0.4	0.0	0.7	0.5	0.3	0.3	0.8	-0.1	0.0	-0.3	0.2	-0.1	0.2	-0.3	0.0	0.3	-

CC4-M		corr_group_3A														corr_group_3B															
corr_group_3A	CD180	-																													
	GPBAR1	0.8	-																												
	TRAF6	0.7	0.6	-																											
	IL10	0.7	0.7	0.7	-																										
	CD14	0.6	0.7	0.5	0.8	-																									
	CD36	0.7	0.5	0.6	0.4	0.2	-																								
	TNFSF13B	0.7	0.7	0.6	0.6	0.4	0.5	-																							
	TCEAL1	0.7	0.7	0.7	0.6	0.5	0.5	0.7	-																						
	DCAF10	0.6	0.5	0.7	0.6	0.5	0.4	0.5	0.5	-																					
	TLR4	0.7	0.5	0.6	0.4	0.3	0.8	0.7	0.7	0.4	-																				
	CPM	0.6	0.5	0.4	0.3	0.3	0.5	0.7	0.6	0.5	0.6	-																			
	CASP4	0.6	0.6	0.6	0.8	0.7	0.2	0.3	0.5	0.6	0.1	0.1	-																		
	IRAK4	0.5	0.5	0.6	0.6	0.6	0.3	0.3	0.5	0.5	0.2	0.2	0.7	-																	
	CASP1	0.7	0.6	0.6	0.6	0.5	0.3	0.4	0.5	0.5	0.3	0.3	0.8	0.7	-																
corr_group_3B	SERPINE1	-0.8	-0.6	-0.7	-0.7	-0.4	-0.6	-0.7	-0.7	-0.4	-0.6	-0.6	-0.4	-0.4	-0.6	-															
	NFKB2	-0.8	-0.6	-0.7	-0.7	-0.5	-0.6	-0.5	-0.7	-0.5	-0.5	-0.5	-0.6	-0.6	-0.6	0.8	-														
	TRAF2	-0.6	-0.5	-0.6	-0.7	-0.4	-0.6	-0.4	-0.6	-0.5	-0.5	-0.4	-0.5	-0.4	-0.5	0.7	0.8	-													
	TRAF1	-0.7	-0.5	-0.5	-0.7	-0.6	-0.5	-0.5	-0.6	-0.4	-0.5	-0.5	-0.4	-0.5	-0.5	0.7	0.8	0.8	-												
	ACE	-0.8	-0.6	-0.7	-0.6	-0.3	-0.7	-0.6	-0.7	-0.5	-0.6	-0.5	-0.4	-0.4	-0.6	0.9	0.8	0.7	0.6	-											
	TNFAIP2	-0.6	-0.3	-0.6	-0.6	-0.4	-0.5	-0.3	-0.6	-0.5	-0.3	-0.5	-0.6	-0.6	-0.7	0.6	0.9	0.7	0.7	0.6	-										
	IL7R	-0.7	-0.6	-0.6	-0.5	-0.3	-0.6	-0.7	-0.5	-0.3	-0.6	-0.5	-0.2	-0.4	-0.4	0.8	0.6	0.6	0.6	0.6	0.4	-									
	TNFSF14	-0.5	-0.6	-0.4	-0.6	-0.5	-0.4	-0.4	-0.5	-0.2	-0.5	-0.2	-0.4	-0.4	-0.3	0.6	0.6	0.6	0.7	0.4	0.4	0.5	-								
	HSP90AB1	-0.5	-0.5	-0.5	-0.6	-0.4	-0.4	-0.3	-0.4	-0.2	-0.2	-0.3	-0.5	-0.5	-0.5	0.6	0.6	0.7	0.6	0.6	0.6	0.6	0.5	-							
	ITGAM	-0.6	-0.6	-0.5	-0.6	-0.5	-0.4	-0.2	-0.5	-0.3	-0.3	-0.1	-0.6	-0.5	-0.5	0.5	0.7	0.7	0.5	0.6	0.6	0.4	0.6	0.7	-						
	CSF1	-0.6	-0.6	-0.5	-0.7	-0.5	-0.5	-0.5	-0.5	-0.2	-0.5	-0.1	-0.4	-0.5	-0.4	0.6	0.6	0.6	0.7	0.4	0.4	0.6	0.8	0.6	0.6	-					
	CCL24	-0.6	-0.6	-0.5	-0.6	-0.5	-0.5	-0.4	-0.5	-0.3	-0.4	-0.2	-0.5	-0.5	-0.4	0.5	0.5	0.5	0.5	0.4	0.4	0.5	0.7	0.5	0.7	0.8	-				
	CD274	-0.6	-0.6	-0.5	-0.6	-0.5	-0.5	-0.4	-0.4	-0.3	-0.5	-0.1	-0.3	-0.4	-0.2	0.4	0.4	0.4	0.3	0.3	0.2	0.4	0.6	0.5	0.5	0.7	0.8	-			
	IL17RA	0.0	0.0	0.1	0.0	0.1	-0.1	0.0	-0.1	0.1	0.1	0.2	0.0	0.0	0.0	0.0	0.0	0.0	-0.1	0.2	0.0	-0.3	0.2	-0.1	0.2	0.0	0.3	0.2	-		
	MMP9	-0.7	-0.5	-0.6	-0.5	-0.4	-0.6	-0.4	-0.4	-0.4	-0.3	-0.5	-0.5	-0.6	-0.5	0.6	0.7	0.5	0.6	0.5	0.7	0.6	0.3	0.7	0.3	0.5	0.4	0.3	-0.2	-	
	IKBKB	-0.6	-0.3	-0.5	-0.5	-0.4	-0.4	-0.3	-0.6	-0.5	-0.3	-0.5	-0.5	-0.4	-0.6	0.6	0.8	0.7	0.5	0.7	0.9	0.3	0.2	0.5	0.5	0.2	0.3	0.1	0.0	0.5	-

CC4-S		corr_group_3A														corr_group_3B															
corr_group_3A	CD180	-																													
	GPBAR1	0.7	-																												
	TRAF6	0.2	0.1	-																											
	IL10	0.7	0.6	0.2	-																										
	CD14	0.7	0.8	-0.2	0.6	-																									
	CD36	-0.1	-0.2	0.5	-0.1	-0.4	-																								
	TNFSF13B	0.7	0.7	0.3	0.6	0.6	0.1	-																							
	TCEAL1	0.5	0.4	0.7	0.4	0.2	0.4	0.6	-																						
	DCAF10	0.1	-0.1	0.6	0.1	-0.3	0.7	0.1	0.5	-																					
	TLR4	0.4	0.3	0.7	0.3	0.0	0.5	0.6	0.7	0.6	-																				
	CPM	0.3	0.0	0.3	0.3	0.2	0.2	0.3	0.3	0.3	0.3	-																			
	CASP4	0.3	0.3	0.1	0.6	0.4	-0.2	0.2	0.4	-0.1	-0.1	0.2	-																		
	IRAK4	0.0	0.1	0.3	0.1	0.0	0.1	0.0	0.4	0.2	0.3	0.0	0.2	-																	
	CASP1	0.5	0.3	0.3	0.6	0.3	-0.1	0.4	0.7	0.0	0.3	0.3	0.7	0.4	-																
corr_group_3B	SERPINE1	-0.7	-0.7	0.0	-0.6	-0.5	0.0	-0.7	-0.3	0.1	-0.4	0.1	-0.2	0.0	-0.4	-															
	NFKB2	-0.4	-0.5	-0.5	-0.6	-0.4	-0.2	-0.4	-0.5	-0.3	-0.4	-0.2	-0.5	-0.4	-0.5	0.3	-														
	TRAF2	-0.3	-0.4	-0.4	-0.3	-0.2	0.0	-0.3	-0.4	0.0	-0.3	-0.3	-0.4	-0.2	-0.4	0.2	0.6	-													
	TRAF1	-0.6	-0.4	-0.2	-0.7	-0.4	0.0	-0.6	-0.4	-0.2	-0.4	-0.3	-0.3	-0.2	-0.4	0.5	0.6	0.3	-												
	ACE	-0.5	-0.3	-0.4	-0.3	-0.1	-0.2	-0.5	-0.5	-0.2	-0.4	-0.2	-0.1	0.0	-0.4	0.4	0.2	0.1	0.2	-											
	TNFAIP2	0.3	0.3	-0.3	0.1	0.2	-0.1	0.3	0.0	-0.1	0.1	-0.2	-0.2	-0.2	-0.1	-0.4	0.4	0.4	-0.1	-0.1	-										
	IL7R	-0.6	-0.6	0.0	-0.4	-0.4	-0.2	-0.6	-0.2	-0.1	-0.4	0.1	0.2	0.2	0.0	0.8	0.1	-0.1	0.4	0.4	-0.5	-									
	TNFSF14	-0.7	-0.7	0.0	-0.7	-0.6	0.3	-0.5	-0.2	0.1	-0.3	0.0	-0.3	-0.1	-0.3	0.7	0.5	0.4	0.6	0.0	-0.2	0.5	-								
	HSP90AB1	0.0	-0.2	0.6	0.0	-0.4	0.7	0.1	0.5	0.8	0.5	0.4	-0.1	0.3	0.1	0.1	-0.3	0.0	-0.3	-0.2	-0.2	0.1	0.2	-							
	ITGAM	0.0	-0.2	-0.1	-0.2	-0.1	0.2	0.2	0.0	0.2	0.1	0.1	-0.4	-0.2	-0.2	-0.1	0.4	0.6	-0.1	-0.3	0.4	-0.3	0.3	0.3	-						
	CSF1	-0.6	-0.6	0.1	-0.6	-0.6	0.4	-0.5	-0.1	0.2	-0.2	0.1	-0.3	-0.1	-0.3	0.7	0.3	0.2	0.6	0.0	-0.3	0.5	0.9	0.3	0.2	-					
	CCL24	-0.6	-0.6	0.0	-0.5	-0.4	0.2	-0.5	-0.2	0.2	-0.2	-0.1	-0.2	0.2	-0.3	0.6	0.1	0.3	0.2	0.5	-0.2	0.5	0.5	0.3	0.2	0.5	-				
	CD274	-0.6	-0.6	-0.1	-0.4	-0.4	0.1	-0.5	-0.2	0.0	-0.5	0.1	0.2	0.0	0.0	0.5	0.1	0.1	0.3	0.2	-0.5	0.6	0.6	0.1	0.1	0.5	0.4	-			
	IL17RA	-0.1	-0.1	0.0	-0.5	-0.2	0.0	0.0	-0.1	0.1	-0.1	-0.1	-0.4	0.0	-0.3	0.3	0.3	0.1	0.3	0.0	0.0	0.1	0.3	0.0	0.4	0.2	0.3	0.1	-		
	MMP9	-0.1	-0.1	-0.2	-0.1	-0.1	-0.1	-0.3	-0.2	-0.1	-0.2	-0.1	-0.2	-0.3	-0.3	0.1	0.2	-0.2	0.2	0.2	-0.1	0.1	0.0	-0.2	-0.2	0.1	0.0	0.1	0.2	-	
	IKBKB	0.3	0.3	-0.3	0.1	0.3	-0.1	0.3	0.1	-0.2	0.1	-0.3	-0.2	0.0	-0.1	-0.4	0.2	0.3	-0.2	0.1	0.8	-0.5	-0.4	-0.1	0.2	-0.5	-0.2	-0.6	-0.1	-0.1	-

macrophages

**control**		CXCL8	CCL3	NCF1	IL1B	CXCL2	TNFAIP3	CCL3L3	CXCL1	TNFAIP6	TNF	CLEC4E
		corr_group_4										
corr_group_4	CXCL8	-										
	CCL3	0.4	-									
	NCF1	0.5	-0.2	-								
	IL1B	0.6	0.3	0.3	-							
	CXCL2	0.7	0.1	0.5	0.6	-						
	TNFAIP3	0.3	0.5	0.2	0.2	-0.1	-					
	CCL3L3	0.1	0.6	-0.4	0.2	-0.1	0.2	-				
	CXCL1	0.2	0.3	0.0	0.4	0.6	-0.3	0.2	-			
	TNFAIP6	0.4	0.2	0.3	0.5	0.6	-0.2	0.1	0.6	-		
	TNF	0.6	0.4	0.2	0.7	0.8	0.0	0.1	0.7	0.7	-	
	CLEC4E	0.7	0.6	0.2	0.2	0.1	0.5	0.4	-0.1	0.0	0.2	-

**control**		IL1RN	PLAUR	TIMP3	TNFAIP8L3	MMP7	IL1R1
		corr_group_5					
corr_group_5	IL1RN	-					
	PLAUR	0.6	-				
	TIMP3	0.9	0.6	-			
	TNFAIP8L3	0.7	0.4	0.7	-		
	MMP7	0.7	0.5	0.6	0.4	-	
	IL1R1	0.5	0.4	0.4	0.1	0.3	-

**CC4-M**		corr_group_4										
corr_group_4	CXCL8	-										
	CCL3	0.8	-									
	NCF1	0.7	0.5	-								
	IL1B	0.8	0.6	0.4	-							
	CXCL2	0.8	0.6	0.5	0.5	-						
	TNFAIP3	0.7	0.5	0.7	0.5	0.3	-					
	CCL3L3	0.6	0.7	0.3	0.7	0.7	0.2	-				
	CXCL1	0.8	0.6	0.4	0.8	0.7	0.4	0.7	-			
	TNFAIP6	0.7	0.6	0.8	0.4	0.6	0.5	0.4	0.5	-		
	TNF	0.5	0.6	0.7	0.3	0.6	0.5	0.5	0.4	0.7	-	
	CLEC4E	0.7	0.5	0.7	0.4	0.3	0.6	0.3	0.3	0.5	0.3	-

**CC4-M**		corr_group_5					
corr_group_5	IL1RN	-					
	PLAUR	0.5	-				
	TIMP3	0.8	0.3	-			
	TNFAIP8L3	0.8	0.4	0.7	-		
	MMP7	0.4	0.3	0.4	0.2	-	
	IL1R1	0.6	0.6	0.3	0.5	0.0	-

**CC4-S**		corr_group_4										
corr_group_4	CXCL8	-										
	CCL3	0.5	-									
	NCF1	0.3	0.3	-								
	IL1B	0.7	0.3	0.7	-							
	CXCL2	0.6	0.2	0.5	0.8	-						
	TNFAIP3	0.6	0.5	0.5	0.5	0.3	-					
	CCL3L3	0.3	0.6	-0.2	-0.1	-0.1	0.3	-				
	CXCL1	0.5	0.2	0.3	0.5	0.7	0.1	0.0	-			
	TNFAIP6	0.5	0.5	0.5	0.4	0.4	0.4	0.1	0.5	-		
	TNF	0.7	0.6	0.3	0.4	0.6	0.2	0.4	0.5	0.6	-	
	CLEC4E	0.8	0.5	0.6	0.7	0.5	0.8	0.2	0.2	0.5	0.4	-

**CC4-S**		corr_group_5					
corr_group_5	IL1RN	-					
	PLAUR	0.6	-				
	TIMP3	0.9	0.6	-			
	TNFAIP8L3	0.5	0.5	0.7	-		
	MMP7	0.7	0.3	0.6	0.3	-	
	IL1R1	0.7	0.5	0.6	0.5	0.7	-

macrophages + IFNγ

control		corr_group_6A																			corr_group_6B																							
		IL10	TNFSF13B	CASP1	SOCS1	IFNGR2	CXCL10	C1QBP	CD180	CASP4	TNFRSF1A	CD36	MYD88	CD14	SERPINB9	IFNGR1	MAFB	STAT1	TLR4	CXCL11	PLAUR	REL	IL1RN	TRAF3IP2	TNFSF14	TIMP3	IL6R	EPAS1	ADAM17	SERPINE1	CSF1	CCL3L3	CCL3	TRAF1	TNFAIP8L3	CXCL8	ARRB2	IRAK2	MMP7	CPM	IL17RA	VEGFA	ITGAM	CD274
	IL10	-																																									
	TNFSF13B	0.7	-																																								
	CASP1	0.8	0.8	-																																							
	SOCS1	0.8	0.8	0.9	-																																						
	IFNGR2	0.6	0.8	0.8	0.7	-																																					
	CXCL10	0.5	0.8	0.8	0.8	0.9	-																																				
	C1QBP	0.6	0.7	0.6	0.7	0.5	0.5	-																																			
	CD180	0.7	0.5	0.6	0.6	0.4	0.3	0.5	-																																		
	CASP4	0.7	0.7	0.8	0.9	0.5	0.6	0.5	0.7	-																																	
corr_group_6A	TNFRSF1A	0.4	0.6	0.4	0.4	0.5	0.4	0.1	0.4	0.3	-																																
	CD36	0.5	0.6	0.4	0.4	0.5	0.4	0.6	0.3	0.2	0.4	-																															
	MYD88	0.7	0.7	0.7	0.8	0.5	0.6	0.8	0.4	0.7	0.2	0.7	-																														
	CD14	0.6	0.7	0.6	0.6	0.6	0.6	0.3	0.5	0.7	0.6	0.4	0.5	-																													
	SERPINB9	0.4	0.4	0.6	0.5	0.7	0.7	0.1	0.3	0.3	0.6	0.1	0.2	0.5	-																												
	IFNGR1	0.7	0.5	0.7	0.5	0.5	0.5	0.6	0.3	0.4	0.3	0.6	0.7	0.4	0.3	-																											
	MAFB	0.6	0.8	0.6	0.6	0.8	0.7	0.4	0.6	0.5	0.7	0.6	0.5	0.7	0.6	0.4	-																										
	STAT1	0.4	0.7	0.6	0.6	0.8	0.7	0.6	0.3	0.6	0.3	0.5	0.6	0.6	0.3	0.5	0.6	-																									
	TLR4	0.2	0.4	0.3	0.2	0.7	0.6	0.1	0.1	0.0	0.5	0.4	0.2	0.2	0.5	0.4	0.6	0.4	-																								
	CXCL11	0.1	0.5	0.4	0.4	0.8	0.8	0.1	0.1	0.3	0.4	0.1	0.1	0.5	0.7	0.1	0.6	0.6	0.6	-																							
	PLAUR	-0.7	-0.8	-0.8	-0.7	-0.8	-0.9	-0.5	-0.4	-0.6	-0.5	-0.5	-0.6	-0.7	-0.7	-0.7	-0.7	-0.7	-0.6	-0.7	-																						
	REL	-0.5	-0.7	-0.6	-0.6	-0.7	-0.7	-0.4	-0.3	-0.5	-0.6	-0.5	-0.4	-0.7	-0.5	-0.6	-0.6	-0.6	-0.6	-0.6	0.8	-																					
	IL1RN	-0.7	-0.7	-0.8	-0.7	-0.7	-0.8	-0.4	-0.4	-0.6	-0.5	-0.4	-0.6	-0.6	-0.6	-0.8	-0.6	-0.5	-0.5	-0.4	0.9	0.8	-																				
	TRAF3IP2	-0.6	-0.7	-0.8	-0.7	-0.8	-0.9	-0.4	-0.5	-0.6	-0.6	-0.4	-0.5	-0.6	-0.7	-0.6	-0.7	-0.6	-0.7	-0.7	0.9	0.8	0.9	-																			
	TNFSF14	-0.6	-0.8	-0.7	-0.7	-0.9	-0.8	-0.5	-0.4	-0.6	-0.6	-0.5	-0.6	-0.8	-0.6	-0.5	-0.8	-0.8	-0.6	-0.8	0.8	0.8	0.7	0.8	-																		
	TIMP3	-0.4	-0.5	-0.6	-0.4	-0.5	-0.6	-0.3	-0.2	-0.4	-0.4	-0.3	-0.4	-0.5	-0.4	-0.5	-0.4	-0.3	-0.5	-0.4	0.7	0.6	0.7	0.7	0.5	-																	
	IL6R	-0.6	-0.6	-0.7	-0.6	-0.7	-0.7	-0.5	-0.2	-0.4	-0.1	-0.4	-0.6	-0.4	-0.5	-0.7	-0.5	-0.5	-0.5	-0.4	0.8	0.5	0.8	0.7	0.5	0.5	-																
	EPAS1	-0.5	-0.6	-0.7	-0.6	-0.8	-0.8	-0.2	-0.3	-0.4	-0.5	-0.3	-0.4	-0.6	-0.7	-0.5	-0.6	-0.6	-0.6	-0.7	0.9	0.7	0.8	0.9	0.8	0.6	0.7	-															
	ADAM17	-0.8	-0.8	-0.7	-0.7	-0.7	-0.7	-0.5	-0.4	-0.6	-0.5	-0.6	-0.6	-0.7	-0.4	-0.8	-0.6	-0.6	-0.4	-0.3	0.8	0.8	0.9	0.8	0.7	0.6	0.7	0.7	-														
	SERPINE1	-0.6	-0.8	-0.8	-0.8	-0.9	-0.8	-0.6	-0.4	-0.7	-0.5	-0.5	-0.7	-0.7	-0.5	-0.6	-0.7	-0.8	-0.4	-0.6	0.8	0.7	0.7	0.7	0.9	0.4	0.6	0.7	0.8	-													
	CSF1	-0.4	-0.7	-0.6	-0.6	-0.8	-0.8	-0.4	-0.4	-0.5	-0.6	-0.4	-0.4	-0.7	-0.5	-0.3	-0.7	-0.7	-0.6	-0.8	0.7	0.7	0.6	0.8	0.9	0.4	0.4	0.7	0.6	0.8	-												
corr_group_6B	CCL3L3	-0.5	-0.7	-0.6	-0.7	-0.6	-0.6	-0.5	-0.4	-0.6	-0.4	-0.3	-0.5	-0.5	-0.2	-0.5	-0.5	-0.5	-0.4	-0.4	0.7	0.7	0.7	0.6	0.6	0.6	0.5	0.5	0.7	0.7	0.6	-											
	CCL3	-0.7	-0.8	-0.7	-0.7	-0.7	-0.6	-0.6	-0.6	-0.6	-0.5	-0.5	-0.6	-0.5	-0.4	-0.7	-0.6	-0.6	-0.4	-0.3	0.7	0.8	0.8	0.7	0.7	0.6	0.6	0.6	0.8	0.7	0.6	0.8	-										
	TRAF1	-0.7	-0.8	-0.8	-0.8	-0.8	-0.8	-0.7	-0.4	-0.7	-0.4	-0.6	-0.7	-0.7	-0.4	-0.7	-0.7	-0.8	-0.5	-0.5	0.9	0.8	0.8	0.8	0.9	0.6	0.6	0.7	0.8	0.9	0.8	0.7	0.8	-									
	TNFAIP8L3	-0.6	-0.7	-0.8	-0.6	-0.7	-0.7	-0.4	-0.5	-0.7	-0.4	-0.3	-0.6	-0.6	-0.5	-0.5	-0.6	-0.6	-0.4	-0.5	0.7	0.7	0.7	0.8	0.7	0.6	0.5	0.6	0.6	0.7	0.6	0.5	0.6	0.7	-								
	CXCL8	-0.6	-0.7	-0.7	-0.6	-0.7	-0.6	-0.4	-0.5	-0.6	-0.6	-0.5	-0.5	-0.7	-0.5	-0.5	-0.7	-0.6	-0.4	-0.4	0.7	0.6	0.5	0.7	0.8	0.4	0.4	0.6	0.6	0.7	0.7	0.5	0.6	0.7	0.6	-							
	ARRB2	-0.2	-0.3	-0.5	-0.3	-0.7	-0.7	-0.1	0.1	-0.2	-0.2	-0.1	-0.2	-0.3	-0.5	-0.4	-0.3	-0.6	-0.6	-0.7	0.7	0.6	0.6	0.7	0.6	0.6	0.5	0.7	0.4	0.6	0.6	0.5	0.3	0.6	0.5	0.3	-						
	IRAK2	-0.7	-0.7	-0.6	-0.6	-0.5	-0.4	-0.6	-0.5	-0.5	-0.4	-0.6	-0.6	-0.5	-0.2	-0.7	-0.6	-0.6	-0.4	-0.2	0.6	0.7	0.7	0.5	0.5	0.3	0.6	0.4	0.7	0.6	0.5	0.6	0.8	0.6	0.5	0.7	0.1	-					
	MMP7	-0.6	-0.7	-0.7	-0.7	-0.7	-0.8	-0.4	-0.4	-0.6	-0.4	-0.4	-0.5	-0.7	-0.5	-0.5	-0.6	-0.6	-0.5	-0.6	0.9	0.7	0.7	0.8	0.8	0.7	0.7	0.8	0.7	0.7	0.7	0.6	0.6	0.8	0.7	0.7	0.5	0.5	-				
	CPM	-0.4	-0.5	-0.5	-0.3	-0.7	-0.6	0.0	-0.2	-0.2	-0.6	-0.3	-0.2	-0.5	-0.7	-0.3	-0.6	-0.3	-0.7	-0.6	0.6	0.6	0.6	0.7	0.6	0.6	0.5	0.8	0.5	0.4	0.5	0.4	0.5	0.4	0.4	0.5	0.5	0.4	0.5	-			
	IL17RA	-0.4	-0.6	-0.6	-0.5	-0.8	-0.7	-0.2	-0.3	-0.5	-0.5	-0.2	-0.4	-0.6	-0.7	-0.4	-0.6	-0.6	-0.4	-0.7	0.7	0.6	0.6	0.7	0.7	0.4	0.5	0.8	0.6	0.7	0.6	0.4	0.4	0.7	0.7	0.5	0.7	0.2	0.6	0.6	-		
	VEGFA	-0.4	-0.5	-0.5	-0.5	-0.6	-0.6	-0.4	-0.3	-0.4	-0.4	-0.4	-0.4	-0.4	-0.5	-0.3	-0.6	-0.5	-0.3	-0.5	0.6	0.4	0.4	0.5	0.6	0.2	0.4	0.5	0.5	0.6	0.6	0.2	0.4	0.5	0.3	0.6	0.2	0.4	0.6	0.4	0.3	-	
	ITGAM	-0.5	-0.7	-0.6	-0.6	-0.6	-0.7	-0.3	-0.3	-0.5	-0.4	-0.3	-0.4	-0.6	-0.5	-0.4	-0.6	-0.5	-0.4	-0.5	0.7	0.6	0.8	0.6	0.5	0.6	0.6	0.6	0.7	0.6	0.5	0.8	0.6	0.6	0.5	0.3	0.4	0.5	0.6	0.5	0.5	0.3	-
	CD274	-0.4	-0.3	-0.2	-0.2	-0.2	-0.2	0.0	-0.4	-0.2	-0.5	-0.2	-0.1	-0.4	-0.4	0.0	-0.4	0.1	-0.2	-0.1	0.3	0.2	0.3	0.3	0.2	0.2	0.3	0.3	0.3	0.1	0.2	0.0	0.3	0.0	0.1	0.5	-0.2	0.4	0.3	0.6	0.1	0.4	0.2	-

CC4-M		corr_group_6A																			corr_group_6B																							
	IL10	-																																									
	TNFSF13B	0.7	-																																								
	CASP1	0.8	0.7	-																																							
	SOCS1	0.8	0.8	0.8	-																																						
	IFNGR2	0.4	0.8	0.5	0.6	-																																					
	CXCL10	0.4	0.7	0.7	0.7	0.8	-																																				
	C1QBP	0.7	0.8	0.6	0.8	0.7	0.6	-																																			
	CD180	0.8	0.6	0.7	0.7	0.3	0.2	0.6	-																																		
	CASP4	0.7	0.6	0.8	0.7	0.4	0.6	0.5	0.5	-																																	
corr_group_6A	TNFRSF1A	0.7	0.7	0.6	0.5	0.3	0.2	0.4	0.7	0.4	-																																
	CD36	0.7	0.5	0.6	0.4	0.2	0.1	0.5	0.6	0.4	0.6	-																															
	MYD88	0.6	0.8	0.6	0.7	0.6	0.6	0.8	0.4	0.6	0.4	0.5	-																														
	CD14	0.7	0.6	0.7	0.6	0.3	0.4	0.4	0.5	0.8	0.6	0.4	0.4	-																													
	SERPINB9	0.5	0.6	0.6	0.5	0.7	0.8	0.5	0.3	0.5	0.3	0.2	0.5	0.4	-																												
	IFNGR1	0.7	0.7	0.7	0.5	0.5	0.4	0.5	0.5	0.5	0.6	0.6	0.4	0.5	0.4	-																											
	MAFB	0.5	0.6	0.4	0.4	0.5	0.3	0.4	0.5	0.2	0.5	0.6	0.3	0.3	0.4	0.8	-																										
	STAT1	0.4	0.8	0.5	0.6	0.8	0.7	0.6	0.3	0.4	0.3	0.2	0.5	0.3	0.5	0.5	0.4	-																									
	TLR4	0.4	0.7	0.4	0.4	0.7	0.4	0.5	0.4	0.1	0.6	0.4	0.3	0.1	0.4	0.7	0.7	0.5	-																								
	CXCL11	0.1	0.4	0.3	0.4	0.7	0.8	0.3	-0.1	0.3	-0.1	-0.3	0.3	0.1	0.6	0.2	0.1	0.6	0.3	-																							
	PLAUR	-0.7	-0.8	-0.8	-0.8	-0.8	-0.8	-0.6	-0.5	-0.6	-0.6	-0.4	-0.5	-0.6	-0.7	-0.7	-0.6	-0.6	-0.6	-0.5	-																						
	REL	-0.6	-0.8	-0.7	-0.7	-0.8	-0.7	-0.6	-0.5	-0.5	-0.7	-0.4	-0.5	-0.5	-0.7	-0.6	-0.5	-0.7	-0.7	-0.5	0.9	-																					
	IL1RN	-0.7	-0.8	-0.8	-0.8	-0.7	-0.8	-0.6	-0.6	-0.6	-0.6	-0.4	-0.5	-0.6	-0.7	-0.7	-0.6	-0.6	-0.6	-0.5	0.9	0.9	-																				
	TRAF3IP2	-0.5	-0.7	-0.6	-0.6	-0.7	-0.8	-0.4	-0.4	-0.4	-0.5	-0.2	-0.3	-0.4	-0.7	-0.6	-0.5	-0.6	-0.7	-0.6	0.9	0.8	0.9	-																			
	TNFSF14	-0.5	-0.7	-0.7	-0.6	-0.7	-0.8	-0.4	-0.4	-0.5	-0.6	-0.3	-0.3	-0.5	-0.7	-0.6	-0.6	-0.6	-0.7	-0.5	0.9	0.8	0.9	1.0	-																		
	TIMP3	-0.4	-0.6	-0.6	-0.6	-0.6	-0.8	-0.3	-0.3	-0.5	-0.4	-0.1	-0.2	-0.5	-0.6	-0.5	-0.3	-0.5	-0.5	-0.6	0.9	0.8	0.8	0.9	0.9	-																	
	IL6R	-0.5	-0.7	-0.6	-0.6	-0.8	-0.7	-0.6	-0.2	-0.6	-0.4	-0.3	-0.6	-0.5	-0.6	-0.5	-0.3	-0.6	-0.5	-0.5	0.8	0.7	0.7	0.7	0.7	0.7	-																
	EPAS1	-0.5	-0.6	-0.7	-0.6	-0.7	-0.8	-0.4	-0.3	-0.6	-0.5	-0.2	-0.4	-0.6	-0.6	-0.6	-0.3	-0.7	-0.4	-0.5	0.8	0.8	0.7	0.8	0.8	0.8	0.6	-															
	ADAM17	-0.8	-0.7	-0.8	-0.8	-0.6	-0.6	-0.6	-0.6	-0.7	-0.7	-0.4	-0.6	-0.6	-0.5	-0.5	-0.3	-0.5	-0.4	-0.2	0.8	0.8	0.8	0.6	0.7	0.7	0.7	0.7	-														
	SERPINE1	-0.6	-0.7	-0.7	-0.6	-0.7	-0.7	-0.5	-0.5	-0.6	-0.6	-0.2	-0.4	-0.5	-0.6	-0.7	-0.5	-0.7	-0.6	-0.5	0.8	0.8	0.8	0.8	0.8	0.7	0.7	0.8	0.8	-													
	CSF1	-0.5	-0.6	-0.6	-0.5	-0.7	-0.7	-0.4	-0.4	-0.4	-0.5	-0.2	-0.3	-0.5	-0.6	-0.6	-0.5	-0.6	-0.6	-0.5	0.8	0.8	0.8	0.9	1.0	0.9	0.6	0.8	0.6	0.8	-												
corr_group_6B	CCL3L3	-0.6	-0.6	-0.6	-0.6	-0.5	-0.5	-0.4	-0.4	-0.5	-0.6	-0.5	-0.4	-0.6	-0.5	-0.7	-0.7	-0.5	-0.6	-0.3	0.8	0.7	0.8	0.7	0.8	0.7	0.6	0.6	0.6	0.6	0.7	-											
	CCL3	-0.8	-0.8	-0.8	-0.7	-0.6	-0.6	-0.6	-0.7	-0.6	-0.8	-0.7	-0.5	-0.6	-0.5	-0.8	-0.8	-0.6	-0.7	-0.2	0.9	0.8	0.9	0.8	0.8	0.7	0.6	0.7	0.7	0.8	0.8	0.8	-										
	TRAF1	-0.7	-0.8	-0.8	-0.7	-0.7	-0.7	-0.6	-0.5	-0.6	-0.6	-0.6	-0.6	-0.5	-0.5	-0.8	-0.7	-0.6	-0.6	-0.4	0.9	0.8	0.9	0.8	0.8	0.7	0.7	0.7	0.7	0.8	0.8	0.8	0.9	-									
	TNFAIP8L3	-0.5	-0.7	-0.7	-0.7	-0.7	-0.8	-0.5	-0.4	-0.5	-0.5	-0.1	-0.4	-0.4	-0.7	-0.5	-0.4	-0.6	-0.5	-0.6	0.8	0.8	0.9	0.9	0.9	0.8	0.7	0.8	0.7	0.9	0.8	0.6	0.7	0.7	-								
	CXCL8	-0.7	-0.5	-0.6	-0.5	-0.3	-0.2	-0.5	-0.5	-0.4	-0.6	-0.6	-0.5	-0.4	-0.2	-0.6	-0.3	-0.3	-0.3	0.2	0.6	0.5	0.5	0.3	0.3	0.3	0.4	0.4	0.6	0.4	0.3	0.4	0.6	0.6	0.4	-							
	ARRB2	0.0	-0.4	-0.3	-0.2	-0.6	-0.7	-0.1	0.0	-0.1	-0.1	0.2	0.0	-0.1	-0.6	-0.3	-0.3	-0.5	-0.5	-0.8	0.6	0.5	0.6	0.8	0.7	0.7	0.5	0.5	0.2	0.6	0.7	0.4	0.4	0.4	0.7	-0.1	-						
	IRAK2	-0.8	-0.7	-0.7	-0.6	-0.5	-0.3	-0.6	-0.7	-0.5	-0.8	-0.7	-0.5	-0.4	-0.3	-0.8	-0.7	-0.5	-0.7	0.0	0.7	0.7	0.7	0.5	0.5	0.4	0.5	0.4	0.7	0.7	0.5	0.7	0.8	0.8	0.5	0.7	0.1	-					
	MMP7	-0.3	-0.5	-0.4	-0.5	-0.5	-0.6	-0.3	-0.3	-0.3	-0.3	0.0	-0.2	-0.3	-0.5	-0.3	-0.2	-0.5	-0.5	-0.5	0.7	0.6	0.7	0.8	0.7	0.8	0.4	0.6	0.6	0.6	0.8	0.5	0.5	0.5	0.7	0.3	0.5	0.3	-				
	CPM	-0.7	-0.7	-0.7	-0.7	-0.6	-0.7	-0.5	-0.5	-0.7	-0.4	-0.4	-0.6	-0.5	-0.6	-0.4	-0.3	-0.5	-0.4	-0.5	0.7	0.7	0.7	0.5	0.6	0.6	0.7	0.5	0.7	0.6	0.5	0.6	0.6	0.7	0.6	0.5	0.3	0.5	0.4	-			
	IL17RA	-0.3	-0.5	-0.5	-0.5	-0.7	-0.8	-0.4	-0.1	-0.5	-0.1	-0.1	-0.5	-0.2	-0.7	-0.2	-0.1	-0.5	-0.3	-0.8	0.6	0.6	0.6	0.5	0.5	0.6	0.7	0.6	0.4	0.5	0.5	0.3	0.3	0.5	0.6	0.2	0.6	0.2	0.3	0.7	-		
	VEGFA	-0.6	-0.6	-0.5	-0.5	-0.4	-0.2	-0.5	-0.5	-0.4	-0.6	-0.7	-0.5	-0.4	-0.3	-0.6	-0.6	-0.5	-0.5	0.0	0.5	0.5	0.5	0.3	0.4	0.2	0.5	0.3	0.6	0.5	0.3	0.6	0.7	0.6	0.3	0.6	0.0	0.8	0.1	0.5	0.2	-	
	ITGAM	0.0	-0.3	-0.1	-0.3	-0.6	-0.6	-0.2	0.0	-0.1	0.1	0.2	-0.2	-0.1	-0.5	0.0	-0.2	-0.4	-0.3	-0.7	0.4	0.5	0.5	0.6	0.5	0.6	0.4	0.4	0.2	0.3	0.5	0.4	0.2	0.3	0.6	-0.3	0.7	-0.1	0.5	0.4	0.6	-0.1	-
	CD274	-0.9	-0.8	-0.8	-0.7	-0.5	-0.5	-0.7	-0.7	-0.6	-0.7	-0.7	-0.6	-0.7	-0.5	-0.7	-0.5	-0.5	-0.5	-0.1	0.7	0.7	0.7	0.5	0.5	0.4	0.5	0.6	0.7	0.6	0.5	0.5	0.8	0.7	0.5	0.7	0.1	0.7	0.3	0.6	0.4	0.6	0.0	-

CC4-S		corr_group_6A																			corr_group_6B																							
	IL10	-																																									
	TNFSF13B	0.7	-																																								
	CASP1	0.8	0.8	-																																							
	SOCS1	0.7	0.7	0.8	-																																						
	IFNGR2	0.6	0.8	0.8	0.6	-																																					
	CXCL10	0.2	0.6	0.5	0.4	0.9	-																																				
	C1QBP	0.6	0.8	0.7	0.7	0.7	0.4	-																																			
	CD180	0.9	0.7	0.6	0.7	0.4	0.1	0.6	-																																		
	CASP4	0.3	0.3	0.6	0.6	0.4	0.3	0.3	0.2	-																																	
corr_group_6A	TNFRSF1A	0.7	0.7	0.4	0.3	0.4	0.1	0.5	0.7	-0.2	-																																
	CD36	0.7	0.8	0.5	0.5	0.6	0.3	0.7	0.6	0.0	0.6	-																															
	MYD88	0.6	0.6	0.7	0.8	0.5	0.3	0.8	0.5	0.5	0.3	0.5	-																														
	CD14	0.5	0.5	0.6	0.5	0.5	0.4	0.3	0.3	0.8	0.2	0.1	0.5	-																													
	SERPINB9	0.6	0.8	0.8	0.5	0.8	0.6	0.6	0.4	0.3	0.6	0.6	0.5	0.5	-																												
	IFNGR1	0.3	0.4	0.3	0.1	0.4	0.4	0.3	0.2	-0.2	0.2	0.7	0.0	-0.1	0.3	-																											
	MAFB	0.5	0.8	0.6	0.5	0.8	0.7	0.5	0.4	0.2	0.4	0.7	0.3	0.3	0.7	0.7	-																										
	STAT1	0.5	0.6	0.5	0.5	0.8	0.7	0.6	0.3	0.1	0.3	0.6	0.4	0.1	0.5	0.7	0.8	-																									
	TLR4	0.4	0.6	0.3	0.1	0.7	0.6	0.4	0.3	-0.3	0.6	0.7	0.0	0.1	0.6	0.7	0.8	0.7	-																								
	CXCL11	0.0	0.4	0.3	0.1	0.7	0.9	0.2	-0.1	0.2	-0.1	0.2	0.1	0.2	0.5	0.4	0.6	0.6	0.6	-																							
	PLAUR	-0.6	-0.7	-0.8	-0.7	-0.8	-0.7	-0.6	-0.5	-0.5	-0.3	-0.5	-0.6	-0.6	-0.6	-0.4	-0.7	-0.6	-0.4	-0.5	-																						
	REL	-0.6	-0.5	-0.6	-0.6	-0.6	-0.4	-0.5	-0.4	-0.3	-0.5	-0.4	-0.6	-0.5	-0.6	0.0	-0.3	-0.4	-0.3	-0.2	0.6	-																					
	IL1RN	-0.7	-0.6	-0.8	-0.6	-0.7	-0.5	-0.4	-0.5	-0.4	-0.4	-0.4	-0.4	-0.5	-0.7	-0.5	-0.6	-0.5	-0.4	-0.3	0.9	0.6	-																				
	TRAF3IP2	-0.6	-0.6	-0.7	-0.6	-0.8	-0.7	-0.5	-0.4	-0.2	-0.4	-0.4	-0.4	-0.4	-0.7	-0.4	-0.6	-0.7	-0.6	-0.5	0.8	0.6	0.8	-																			
	TNFSF14	-0.5	-0.7	-0.7	-0.7	-0.9	-0.7	-0.6	-0.4	-0.4	-0.3	-0.5	-0.6	-0.5	-0.7	-0.3	-0.6	-0.7	-0.5	-0.6	0.9	0.7	0.7	0.8	-																		
	TIMP3	-0.1	-0.2	-0.2	0.1	-0.1	-0.1	0.2	-0.1	-0.1	-0.1	0.2	0.3	-0.3	-0.2	-0.2	-0.2	0.1	-0.2	-0.1	0.2	-0.1	0.4	0.2	-0.1	-																	
	IL6R	-0.4	-0.4	-0.6	-0.5	-0.7	-0.7	-0.4	-0.2	-0.5	0.0	-0.1	-0.3	-0.6	-0.5	-0.3	-0.5	-0.5	-0.3	-0.5	0.8	0.4	0.8	0.7	0.7	0.4	-																
	EPAS1	-0.3	-0.4	-0.5	-0.4	-0.5	-0.4	-0.2	-0.2	-0.6	-0.1	0.0	-0.4	-0.7	-0.4	0.0	-0.3	-0.1	-0.1	-0.3	0.7	0.5	0.7	0.5	0.5	0.3	0.6	-															
	ADAM17	-0.6	-0.6	-0.8	-0.7	-0.7	-0.4	-0.6	-0.5	-0.6	-0.3	-0.3	-0.7	-0.6	-0.6	-0.1	-0.3	-0.4	-0.1	-0.2	0.8	0.7	0.8	0.6	0.7	0.1	0.7	0.6	-														
	SERPINE1	-0.5	-0.6	-0.6	-0.6	-0.7	-0.6	-0.4	-0.4	-0.4	-0.3	-0.4	-0.5	-0.5	-0.5	-0.3	-0.5	-0.6	-0.4	-0.4	0.8	0.6	0.7	0.8	0.8	0.0	0.6	0.6	0.7	-													
	CSF1	-0.5	-0.6	-0.6	-0.6	-0.8	-0.7	-0.5	-0.4	-0.3	-0.4	-0.4	-0.4	-0.5	-0.6	-0.3	-0.6	-0.7	-0.6	-0.6	0.7	0.7	0.6	0.9	0.9	0.0	0.6	0.5	0.5	0.8	-												
corr_group_6B	CCL3L3	-0.4	-0.4	-0.5	-0.4	-0.3	-0.2	-0.1	-0.4	-0.2	-0.2	-0.1	-0.1	-0.3	-0.3	-0.3	-0.4	-0.3	-0.2	-0.1	0.4	0.1	0.4	0.3	0.2	0.5	0.4	0.1	0.2	0.1	0.2	-											
	CCL3	-0.8	-0.7	-0.8	-0.6	-0.7	-0.5	-0.5	-0.6	-0.3	-0.5	-0.6	-0.5	-0.5	-0.6	-0.6	-0.7	-0.6	-0.5	-0.2	0.8	0.6	0.9	0.8	0.7	0.3	0.6	0.5	0.7	0.7	0.7	0.4	-										
	TRAF1	-0.6	-0.7	-0.8	-0.8	-0.8	-0.7	-0.7	-0.5	-0.4	-0.3	-0.6	-0.7	-0.4	-0.6	-0.5	-0.6	-0.8	-0.4	-0.5	0.9	0.6	0.7	0.7	0.9	0.0	0.7	0.4	0.7	0.7	0.7	0.3	0.7	-									
	TNFAIP8L3	-0.6	-0.5	-0.6	-0.6	-0.6	-0.3	-0.5	-0.4	-0.3	-0.4	-0.3	-0.6	-0.5	-0.5	-0.1	-0.3	-0.4	-0.2	-0.1	0.6	0.8	0.7	0.7	0.7	-0.1	0.5	0.5	0.7	0.7	0.7	0.1	0.6	0.6	-								
	CXCL8	-0.7	-0.6	-0.6	-0.6	-0.7	-0.5	-0.6	-0.5	-0.2	-0.4	-0.6	-0.6	-0.4	-0.5	-0.4	-0.6	-0.6	-0.4	-0.3	0.8	0.8	0.7	0.7	0.8	-0.2	0.5	0.5	0.7	0.6	0.7	0.2	0.8	0.8	0.7	-							
	ARRB2	-0.2	-0.2	-0.4	-0.2	-0.5	-0.6	0.0	-0.1	-0.3	0.0	0.0	0.0	-0.4	-0.4	-0.2	-0.4	-0.3	-0.4	-0.6	0.6	0.3	0.6	0.6	0.5	0.4	0.7	0.5	0.4	0.5	0.6	0.4	0.4	0.4	0.4	0.3	-						
	IRAK2	-0.7	-0.6	-0.6	-0.5	-0.6	-0.4	-0.5	-0.5	-0.1	-0.6	-0.7	-0.5	-0.4	-0.5	-0.4	-0.6	-0.5	-0.5	-0.2	0.5	0.7	0.6	0.6	0.6	0.0	0.4	0.4	0.5	0.4	0.6	0.1	0.7	0.5	0.6	0.8	0.3	-					
	MMP7	0.0	-0.2	-0.2	0.0	-0.5	-0.6	0.0	0.1	-0.2	-0.1	0.1	0.1	-0.4	-0.5	0.0	-0.3	-0.2	-0.3	-0.5	0.4	0.3	0.4	0.5	0.3	0.4	0.5	0.5	0.2	0.5	0.5	0.1	0.3	0.1	0.2	0.1	0.6	0.2	-				
	CPM	-0.5	-0.5	-0.6	-0.4	-0.4	-0.4	-0.2	-0.4	-0.4	-0.3	-0.2	-0.3	-0.5	-0.6	0.0	-0.4	-0.1	-0.2	-0.2	0.4	0.3	0.6	0.4	0.2	0.5	0.4	0.5	0.6	0.4	0.2	0.3	0.4	0.3	0.3	0.2	0.4	0.3	0.4	-			
	IL17RA	-0.1	-0.3	-0.5	-0.4	-0.5	-0.6	-0.2	0.0	-0.6	0.2	0.0	-0.4	-0.5	-0.5	0.2	-0.3	-0.2	0.0	-0.5	0.6	0.4	0.4	0.4	0.6	0.0	0.5	0.6	0.5	0.4	0.5	0.1	0.1	0.5	0.3	0.4	0.5	0.2	0.4	0.5	-		
	VEGFA	-0.5	-0.6	-0.5	-0.5	-0.7	-0.6	-0.4	-0.4	-0.3	-0.3	-0.5	-0.4	-0.4	-0.6	-0.4	-0.5	-0.5	-0.5	-0.5	0.6	0.4	0.6	0.6	0.6	0.2	0.6	0.3	0.5	0.5	0.5	0.4	0.6	0.6	0.4	0.4	0.4	0.4	0.2	0.4	0.3	-	
	ITGAM	-0.2	-0.4	-0.6	-0.5	-0.6	-0.6	-0.2	-0.2	-0.7	0.1	0.1	-0.3	-0.6	-0.5	0.0	-0.4	-0.4	-0.1	-0.5	0.7	0.2	0.6	0.5	0.5	0.4	0.7	0.6	0.5	0.6	0.5	0.4	0.4	0.6	0.3	0.2	0.7	0.0	0.5	0.6	0.7	0.5	-
	CD274	-0.8	-0.7	-0.5	-0.4	-0.6	-0.3	-0.6	-0.6	0.0	-0.8	-0.7	-0.4	-0.3	-0.6	-0.3	-0.5	-0.4	-0.6	-0.1	0.4	0.6	0.5	0.5	0.4	0.1	0.2	0.2	0.4	0.4	0.5	0.2	0.7	0.4	0.4	0.6	0.1	0.8	0.1	0.3	0.0	0.5	0.0	-

macrophages + IFNγ

**control**		corr_group_7							
		JAK2	IRAK3	JAK1	CHUK	IFNAR1	PRCP	CCR5	TNFAIP6
corr_group_7	JAK2	-							
	IRAK3	0.1	-						
	JAK1	0.1	0.3	-					
	CHUK	0.2	0.1	0.1	-				
	IFNAR1	0.0	0.5	0.4	0.3	-			
	PRCP	0.6	-0.1	0.0	0.2	-0.2	-		
	CCR5	0.0	0.3	0.4	0.3	0.5	0.0	-	
	TNFAIP6	0.2	-0.1	-0.1	0.2	0.1	0.4	0.2	-

**CC4-M**		corr_group_7							
corr_group_7	JAK2	-							
	IRAK3	0.3	-						
	JAK1	0.4	0.5	-					
	CHUK	0.5	0.3	0.4	-				
	IFNAR1	0.6	0.6	0.6	0.6	-			
	PRCP	0.3	0.2	0.3	0.4	0.3	-		
	CCR5	0.2	0.4	0.4	0.1	0.3	0.2	-	
	TNFAIP6	0.2	0.4	0.5	0.4	0.5	0.2	0.7	-

**CC4-S**		corr_group_7							
corr_group_7	JAK2	-							
	IRAK3	0.7	-						
	JAK1	0.7	0.7	-					
	CHUK	0.6	0.7	0.7	-				
	IFNAR1	0.7	0.8	0.7	0.6	-			
	PRCP	0.8	0.6	0.6	0.6	0.6	-		
	CCR5	0.5	0.7	0.7	0.7	0.8	0.7	-	
	TNFAIP6	0.3	0.7	0.5	0.5	0.6	0.3	0.8	-
